# *Helicobacter hepaticus* is required for immune targeting of bacterial heat shock protein 60 and fatal colitis in mice

**DOI:** 10.1080/19490976.2021.1882928

**Published:** 2021-02-08

**Authors:** Verena Friedrich, Ignasi Forné, Dana Matzek, Diana Ring, Bastian Popper, Lara Jochum, Stefanie Spriewald, Tobias Straub, Axel Imhof, Anne Krug, Bärbel Stecher, Thomas Brocker

**Affiliations:** a Institute for Immunology, BioMedical Center, Faculty of Medicine, LMU Munich, Munich, Germany; b Protein Analysis Unit, BioMedical Center, Faculty of Medicine, LMU Munich, Munich, Germany; c Core Facility Animal Models, BioMedical Center, Faculty of Medicine, LMU Munich, Munich, Germany; d Max von Pettenkofer Institute of Hygiene and Medical Microbiology, LMU Munich, Munich, Germany; e Core Facility Bioinformatics, BioMedical Center, Faculty of Medicine, LMU Munich, Munich, Germany; f German Center for Infection Research (DZIF), Partner Site, Munich, Germany

**Keywords:** Colitis, CD40, *Helicobacter hepaticus*, GroEL, CD103^+^ DCs, iTregs

## Abstract

Gut microbiota and the immune system are in constant exchange shaping both host immunity and microbial communities. Here, improper immune regulation can cause inflammatory bowel disease (IBD) and colitis. Antibody therapies blocking signaling through the CD40–CD40L axis showed promising results as these molecules are deregulated in certain IBD patients. To better understand the mechanism, we used transgenic DC-LMP1/CD40 animals with a constitutive CD40-signal in CD11c^+^ cells, causing a lack of intestinal CD103^+^ dendritic cells (DCs) and failure to induce regulatory T (iTreg) cells. These mice rapidly develop spontaneous fatal colitis, accompanied by dysbiosis and increased inflammatory IL-17^+^IFN-γ^+^ Th17/Th1 and IFN-γ ^+^ Th1 cells. In the present study, we analyzed the impact of the microbiota on disease development and detected elevated IgA- and IgG-levels in sera from DC-LMP1/CD40 animals. Their serum antibodies specifically bound intestinal bacteria, and by proteome analysis, we identified a 60 kDa chaperonin GroEL (Hsp60) from *Helicobacter hepaticus* (*Hh*) as the main specific antigen targeted in the absence of iTregs. When re-derived to a different *Hh*-free specific-pathogen-free (SPF) microbiota, mice showed few signs of disease, normal microbiota, and no fatality. Upon recolonization of mice with *Hh*, the disease developed rapidly. Thus, the present work identifies GroEL/Hsp60 as a major *Hh*-antigen and its role in disease onset, progression, and outcome in this colitis model. Our results highlight the importance of CD103^+^ DC- and iTreg-mediated immune tolerance to specific pathobionts to maintain healthy intestinal balance.

## Introduction

The large intestine is colonized with about 10^11^–10^12^ bacterial cells/g of luminal content,^1^ to which mucosal immune cells are constantly exposed. These interactions are indispensable to generate tolerance toward harmless commensals or immunity to invading pathogens. The intestinal microbiota has a critical impact on modulating host immune responses in both health and disease.^2–4^ However, multiple genetic and environmental factors such as immune deficiency, infection, inflammation, or antibiotic treatment alter the microbial composition and direct mucosal homeostasis toward dysbiosis. Inflammatory Bowel Disease (IBD) is linked to dysbiosis and many studies revealed altered bacterial composition in IBD patients.^5–7^ However, it remains elusive whether dysbiosis is the cause or rather a consequence of IBD.^8^

IL-17-producing T helper (Th17) cells are not detectable in the intestine of germ-free mice but can be effectively induced in the small intestinal lamina propria (LP) by mono-colonization with segmented filamentous bacteria.^9,10^ Moreover, regulatory T cells (Tregs) are affected by the gut microbiota as Clostridium clusters IV and XIVa are potent drivers of IL-10^+^Helios^−^ induced T regs (iTregs).^11^ These and other reports illustrate how the immune system is shaped by the microbiota of the gut. Similarly, the murine commensal *Helicobacter hepaticus* (*Hh*) is found in many academic and commercial mouse colonies^12,13^ and infection with *Hh* is linked to chronic hepatitis and hepatocellular carcinoma.^14,15^ *Hh* can also elicit intestinal inflammation in immunodeficient mice.^16,17^ Moreover, IL-10- or T cell-deficient mice require *Hh* for the development of colitis.^18–20^ However, there are still major gaps in our understanding of the complex interaction between the microbial community, a certain single species or even bacterial antigens, and the host.

Chaperonins, a subset of molecular chaperones, control proper protein folding and are present in many bacteria (GroEL) and eukaryotic organelles (heat shock protein (Hsp)60). High similarity and molecular mimicry between the bacterial and human ortholog^21^ induce antibodies cross-reacting with Hsp60 of both species,^22^ contributing to IBD and various autoimmune diseases (reviewed in ref. ^23^). Hsp60 has been suggested as a biomarker and potential pathogenic agent in IBD, as it triggers pro-inflammatory cytokines.^23^

We recently published a novel CD40-mediated mouse model of spontaneous colitis, where CD11c-specific constitutive CD40-signaling leads to migration of CD103^+^ DCs from the colonic LP to draining lymph nodes followed by DC-apoptosis.^24^ Loss of tolerogenic CD103^+^ DCs caused a lack of RORγt^+^Helios^−^ iTregs and an increase of inflammatory IL-17^+^IFN-γ^+^ Th17/Th1 and IFN-γ^+^ Th1 cells in the colon, resulting in the breakdown of mucosal tolerance and fatal colitis.^24–26^ Of note, this model mimics the human IBD situation, as CD40-CD40L interactions are of relevance to the pathogenesis of IBD.^27–33^

In the present study, we focused on microbial-host interactions in the CD40-mediated colitis model to determine how the intestinal microbiota modulates the host immune response. We identified *Hh* as a disease driver with an impact on disease onset, progression, and outcome in mice lacking iTregs and CD103^+^ DCs due to CD11c-driven constitutive CD40-signaling. The immune response of diseased animals targets *Hh*, and we identified GroEL, a 60 kDa *Hh*-protein, as the main antigen recognized by antibodies during the onset of fatal colitis. Rederivation of the mice to *Hh*-free state saved mice from fatal colitis. This suggested that *Hh* could trigger colitis and specific immune responses in an iTreg-free setting.

## Results

### Early disease onset in DC-LMP1/CD40 mice is associated with increased serum antibody levels specific for bacterial antigens

DC-LMP1/CD40 mice have been described previously.^24–26^ They were generated by breeding CD11c-Cre mice^34^ to LMP1/CD40^flStop^ mice,^35^ which express a loxP-flanked stop-codon-protected LMP1/CD40 chimeric molecule. This caused constitutive CD40-signaling in CD11c^+^ DCs and as a consequence, the DC-iTreg axis was altered and mice developed fatal colitis.^24^ This signal caused loss of intestinal CD103^+^ DCs, deficiency to generate iTregs and increased IL-17^+^IFNγ^+^ Th17/Th1 cells and pathogenic IFNγ^+^ Th1 cells.^24,25^

**Figure 1.**
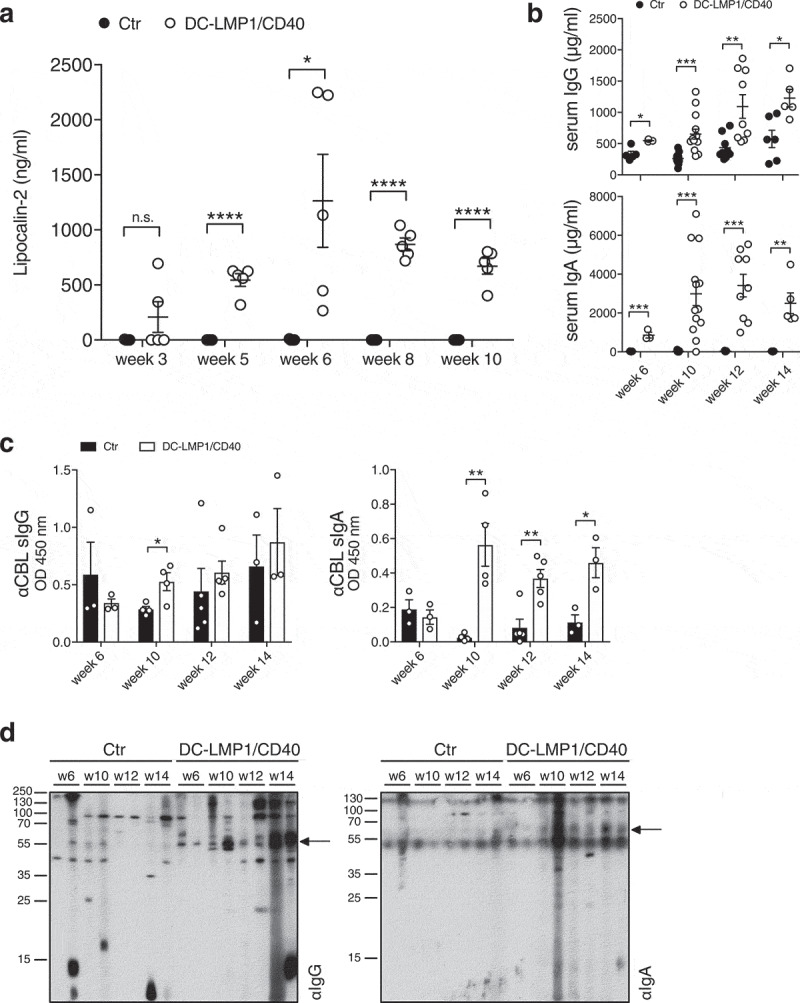
Early disease onset and increasing serum antibody titers. (a) Levels of fecal lipocalin-2 were measured by ELISA in control (Ctr) and DC-LMP1/CD40 mice at the indicated time points. Data are shown as mean ± SEM (n = 5). (b) Total IgG (upper panel) or IgA (lower panel) concentrations in sera from Ctr and DC-LMP1/CD40 mice at the indicated time points were measured by ELISA. Data from two pooled experiments are shown as mean ± SEM (n = 3–13). (c-d) Serum IgG (left) and IgA (right) response of Ctr and DC-LMP1/CD40 mice toward commensal antigens within the CBL was determined by (c) ELISA (mean ± SEM, n = 3–5 per group and time point, one representative experiment of two is shown) or (d) immunoblotting at the indicated time points (n = 2 per group and time point, each lane represents one serum sample from Ctr or DC-LMP1/CD40 mice, arrows indicate a 60 kDa protein). Goat anti-mouse IgG-HRP or goat anti-mouse IgA-HRP were used as secondary antibodies

To get further insight into the complex interplay of microbiota, adaptive immunity and inflammation in CD40-mediated colitis, we first determined the disease onset in DC-LMP1/CD40 mice by measuring fecal lipocalin-2, a sensitive noninvasive inflammatory marker.^36^ Lipocalin-2 levels were significantly increased in DC-LMP1/CD40 mice starting from week 5 (Figure 1(a)), showing a very early disease onset because of constitutive CD40-signaling on DCs as published previously.^24^ To measure a potential impact on adaptive immunity, we analyzed IgG and IgA serum levels during colitis progression. Compared to control littermates, DC-LMP1/CD40 mice showed elevated total serum IgG- as well as IgA-levels already at 6 weeks and increased further with age (Figure 1(b)). As mice with spontaneous colitis have the propensity to develop antibody responses against commensal bacteria,^37^ we next set out to identify antibody specificities in DC-LMP1/CD40 mice. We used cecal bacterial lysate (CBL) from C57BL/6 mice of the same colony, representing unaltered intestinal microbiota for ELISA.^37^ In DC-LMP1/CD40 mice, serum IgG response to commensal antigens was significantly increased at the 10-week time point if compared to control littermates (Figure 1(c), left). In contrast, we detected significantly higher serum IgA reactivities starting at the age of 10 weeks at all time points analyzed (Figure 1(c), right). To further visualize bacterial antigens potentially recognized by serum Ig from DC-LMP1/CD40 mice, we tested these sera also by immunoblotting (Figure 1(d)). Serum IgG from both DC-LMP1/CD40 mice and control littermates, detected some proteins of different sizes ranging from 10 to 250 kDa (Figure 1(d), left). However, in contrast to sera from controls, each serum IgG sample from DC-LMP1/CD40 mice showed reactivity with a protein of about 60 kDa (Figure 1(d), left, arrow). This reactivity increased with the age of mice (Figure 1(d), left). Furthermore, serum IgA from 10-, 12- and 14-week samples of DC-LMP1/CD40, but not control mice, detected proteins around 60 kDa (Figure 1(d), right, arrow). Our data reveal a very early disease onset in DC-LMP1/CD40 mice simultaneously with an increase of serum reactivity against commensal antigens present in CBL from C57BL/6 mice of the same colony.

### Serum antibodies from DC-LMP1/CD40 mice are specific for a 60 kDa chaperonin from Helicobacter hepaticus

**Figure 2.**
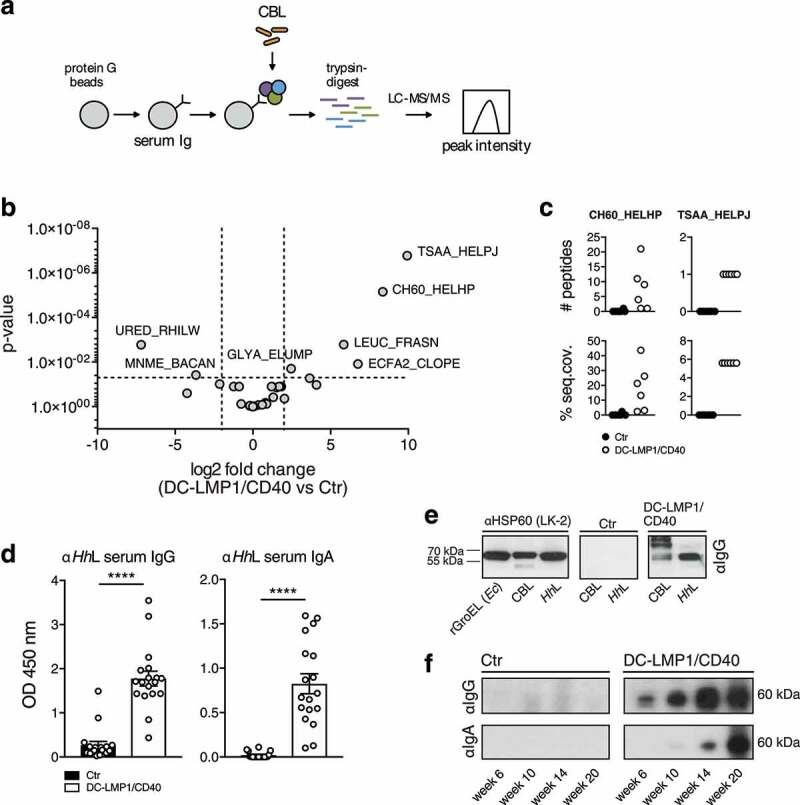
Analysis of fecal antigens. (a) Schematic illustration of sample preparation for liquid chromatography tandem mass spectrometry (LC-MS/MS). Protein G beads were coupled with serum antibodies from Ctr or DC-LMP1/CD40 mice to bind commensal antigens within the CBL. Upon immunoprecipitation, proteins were trypsin-digested, analyzed by LC-MS/MS and the resulting peak intensity was used for intensity-based absolute quantification (iBAQ) (pooled results from two experiments, n = 6, about 40% of serum samples were also included in Figure 1(c)). (b) Results obtained with iBAQ as described in (a) are illustrated by the volcano plot. Identified proteins were considered as interaction partners if the log2 difference between the iBAQ values in the DC-LMP1/CD40 condition and the controls was higher than 2 and the *p*-value smaller than 0.05 (ANOVA). (c) Data illustrates the number of peptides (upper panel) and the percentage of protein sequence coverage (lower panel) of the identified CH60_HELHP and TSAA_HELPJ in (b). Each symbol represents one single mouse. (d) Serum IgG (left) and IgA (right) response of 6- to 20-week-old Ctr and DC-LMP1/CD40 mice toward lysate from *Hh* (*Hh*L) was determined by ELISA (mean ± SEM, n = 18, one representative experiment of two is shown, including all sera that were also tested in Figure 1(c)). (e) Detection of the 60 kDa protein in *Hh*L and CBL by immunoblotting. 20 µg *Hh*L, 50 µg CBL, or 0.5 µg recombinant GroEL from *E.coli* (rGroEL (*Ec*)) were separated by SDS-PAGE. Anti-HSP60 (left, clone LK-2: recognizing both human and bacterial Hsp60, mouse IgG1 isotype) as well as sera from one 10-week-old Ctr (middle) and one 10-week-old DC-LMP1/CD40 (right) mouse (which were previously tested in (a)) were used as primary antibodies. Anti-mouse IgG-HRP was used as a secondary antibody. (f) Sera screening for the detection of 60 kDa chaperonin from *Hh* by immunoblotting. 200 µg *Hh*L were separated by SDS-PAGE using the Mini-PROTEAN II Multiscreen Apparatus. Sera from Ctr or DC-LMP1/CD40 mice at the indicated age (which were previously tested in (a)) were used as primary antibodies with each lane representing one serum sample. Anti-mouse IgG-HRP (upper panel) or anti-mouse IgA-HRP (lower panel) were used as secondary antibodies

To identify antigens recognized by serum Ig in DC-LMP1/CD40 animals, we performed liquid chromatography tandem mass spectrometry (LC-MS/MS) (Figure 2(a)). For this approach, serum antibodies were coupled to beads and incubated with CBL for binding of potential target proteins. Upon immunoprecipitation, we performed on-bead digestion of proteins followed by LC-MS/MS. The resulting peak intensities were finally used for intensity-based absolute quantification (iBAQ). Proteins identified with a fold change > 2 and a *p*-value < 0.05 were considered for further analysis. Interestingly, the results provided only five proteins precipitated by serum antibodies from DC-LMP1/CD40 mice and two proteins by control serum antibodies (Figure 2(b)) that met these requirements. We focused on proteins precipitated by serum antibodies from DC-LMP1/CD40 animals with the highest fold change and lowest *p*-value. These were the 60 kDa chaperonin GroEL (Hsp60) from *Helicobacter hepaticus* (*Hh*) (CH60_HELHP, 8.36-fold change, *p*-value < 0.00001) and the probable peroxiredoxin from *Helicobacter pylori* (TSAA_HELPJ, 9.93-fold change, *p*-value < 0.000001). The data analysis for the number of precipitated peptides and the percentage of sequence coverage of the protein revealed that the CH60_HELHP was identified by 1–21 peptides with a sequence coverage ranging from 2.4% up to 43.7% (Figure 2(c)). In contrast, TSAA_HELPJ was identified by only one peptide and with a sequence coverage of only 5.6% for every single DC-LMP1/CD40 serum sample (Figure 2(c)). This protein from *H. pylori* was not considered for further analysis as both the number of peptides and the percentage of protein sequence coverage were not reliable. One explanation for recovering a protein from *H. pylori* with this approach might be the fact that about 50% of the total proteins from *Hh* have orthologs in *H. pylori*^38^ and therefore might arise from the analysis within the bacterial database used for iBAQ. Indeed, analysis of the *H. pylori* peptide sequence with BLAST against the *Hh* proteome resulted in 100% identity with peroxiredoxin from *Helicobacter* multispecies as well as 70% identity with chemotaxis protein from *Hh*.

To exclude biased results due to differences in serum antibody amounts from DC-LMP1/CD40 and control animals bound by protein G beads, samples were adjusted by calculating equal amounts of serum IgG before coupling to the beads and the peak intensities of Ig-related proteins were quantified within the same experiment. Here, DC-LMP1/CD40 and control serum samples showed no differences in Ig-related protein intensities (Figure S1), indicating equal coupling of serum Ig from control and transgenic mice.

We next tested serum antibody reactivity from DC-LMP1/CD40 mice toward whole *Hh*-lysate (*Hh*L) by ELISA (Figure 2(d)) and immunoblotting (Figure 2(e)). Indeed, both serum IgG and IgA from DC-LMP1/CD40 mice showed a strong reactivity toward *Hh*L when compared to sera from control littermates by ELISA (Figure 2(d)), corroborating previous reports that mice infected with *Hh* produce *Hh*-specific serum Ig.^39,40^ To detect GroEL from *Hh* by immunoblotting, we used the monoclonal anti-human heat shock protein 60 (αHsp60) antibody (clone LK-2, mouse IgG1 isotype) as a positive control, which specifically recognizes both, human Hsp60 and the bacterial homologue GroEL.^41^ As expected, in Western blot analysis, αHsp60 (LK-2) detected recombinant GroEL from *E. coli* (rGroEL (*Ec*)), from CBL and from *Hh*L (Figure 2(e), left panel), confirming the specificity of this antibody and the presence of GroEL in CBL used for this screening. Furthermore, in contrast to sera from control littermates (Figure 2(e), middle panel), sera from DC-LMP1/CD40 mice (Figure 2(e), right panel) detected a band of the same size in CBL as well as in *Hh*L. Interestingly, we detected GroEL in *Hh*L with serum IgG from DC-LMP1/CD40 animals at every age tested, and this reactivity was increasing with the age of mice (Figure 2(f)). In contrast, GroEL detection from *Hh*L with serum IgA from DC-LMP1/CD40 mice was observed only with sera obtained from mice at the age of 14 weeks and older (Figure 2(f)). However, there was no GroEL-specific signal detected neither with serum IgG nor IgA from control mice (Figure 2(f)), although their fecal content did contain *Hh* (see below). Taken together, we identified the 60 kDa chaperonin GroEL from *Hh* as a potential antigen recognized by the immune system during early colitis onset, indicating that *Hh* could be a disease driver in the DC-LMP1/CD40 colitis model.

### Helicobacter hepaticus-free DC-LMP1/CD40 mice are protected from early disease onset

**Figure 3.**
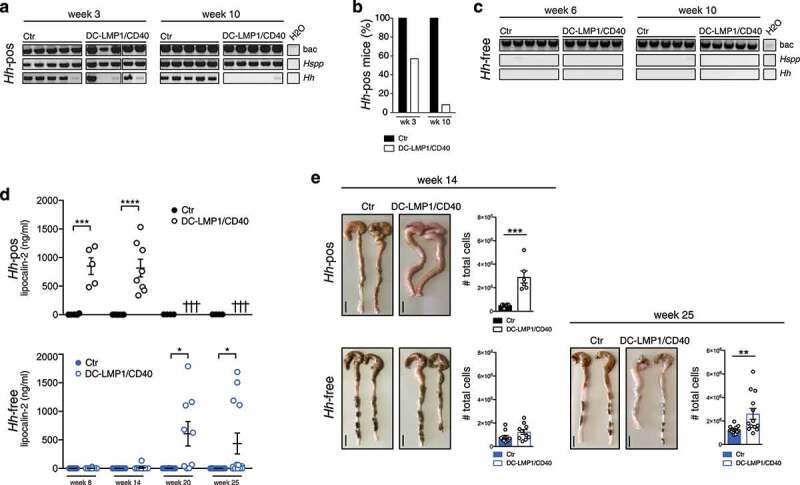
*Hh*-free DC-LMP1/CD40 are protected from early disease onset. (a) Bacterial DNA was extracted from Ctr or DC-LMP1/CD40 mice before rendering them *Hh*-free at the indicated time points. 16S rRNA gene primers were used to detect the species indicated and amplicons were analyzed by gel electrophoresis (n = 7–14, shown are n = 5 per group). (b) Data for *Hh*-pos animals from (a) are represented as bar graphs, illustrating the percentage of 3-or 10-week-old Ctr and DC-LMP1/CD40 animals tested positive for *Hh* before rendering them *Hh*-free (n = 7–14 per group). (c) Bacterial DNA was extracted from Ctr or DC-LMP1/CD40 mice after rendering them *Hh*-free at the indicated time points. 16S rRNA gene primers were used to detect the species indicated and amplicons were analyzed by gel electrophoresis (n = 5–9, shown are n = 5 per group).(d) Levels of fecal lipocalin-2 were measured by ELISA in *Hh*-pos (upper panel) or *Hh*-free (lower panel) Ctr and DC-LMP1/CD40 mice at the indicated time points. Shown are data from two pooled experiments for *Hh*-pos animals (n = 5–9) and for *Hh*-free animals (n = 9–13) as mean ± SEM. Crosses represent already dead animals at the indicated time points. (e) Macroscopic pictures as well as total cell number of colons from *Hh*-pos (upper panel) or *Hh*-free (lower panel) Ctr and DC-LMP1/CD40 animals at the indicated time points. Shown are two representative colon pictures per group with scale bars = 1 cm. Bar graphs show total colon cell numbers in Ctr and DC-LMP1/CD40 mice from three pooled experiments (mean ± SEM, n = 6–13). bac: bacteria; *Hspp: Helicobacter species; Hh: H. hepaticus.*

The intestinal bacterium *Hh* is associated with IBD and induces spontaneous colitis in mice with severe combined immunodeficiency or IL10-deficiency.^16,18^ The fact that we found *Hh*-specific Ig in sera of DC-LMP1/CD40 mice suggested that this mouse colony was endemically infected by *Hh*. To test this, we screened the fecal content from mice for the presence of *Helicobacter* by genus-specific and species-specific PCR (Figure 3(a)). We found the genus *Helicobacter* (*Hspp)* throughout all DC-LMP1/CD40 and control littermates (Figure 3(a)). Moreover, all control littermates were consistently colonized with *Hh* (Figure 3(a)). Surprisingly, young DC-LMP1/CD40 mice showed reduced prevalence already in week 3 of age, when only 57.1% *Hh*-positive transgenic animals could be detected, in contrast to 100% *Hh*-positive control littermates (Figure 3(b)). Furthermore, *Hh* was hardly detectable in older DC-LMP1/CD40 mice, as in 10-week-old animals only 8.3% were *Hh*-positive as compared to 100% of control littermates (Figure 3(b)). Notably, we obtained similar results for colonization with *H. typhlonius* (*Ht*) (Figure S2). In contrast, all animals tested were also colonized by *H. rodentium* (*Hr*), explaining the consistent *Hspp* positive results (Figure S2). None of the animals was tested positive for *H. bilis* (*Hb*) (Figure S2). Taken together, conventionally housed mice were endemically colonized with *Hh*. The fact that DC-LMP1/CD40 animals show loss of *Hh* colonization, in particular upon colitis progression, suggests that these bacteria are eliminated by either ongoing immune responses or displacement by other bacteria during dysbiosis. To ultimately identify *Hh* as a disease driver in this model of colitis, we rederived mice to an *Hh*-free specific-pathogen-free (SPF) colony by embryo transfer. *Hh-*colonization status was confirmed by genus- and species-specific PCR with fecal content from 6- and 10-week-old mice (Figure 3(c)). Notably, all animals were tested negative for *Hh* (Figure 3(c)) as well as *Ht, Hr*, and *Hb* (Figure S2).

None of the *Hh*-free DC-LMP1/CD40 animals showed elevated fecal lipocalin-2 levels at the age of 8 or 14 weeks, when *Hh*-positive (*Hh-pos*) DC-LMP1/CD40 mice had already significantly elevated fecal lipocalin-2 levels (Figure 3(d)). Interestingly, we did detect significantly increased fecal lipocalin levels only at much later time points in some but not all *Hh*-free DC-LMP1/CD40 mice (Figure 3(d)). When we compared the phenotype of *Hh*-pos and *Hh*-free animals at the age of 14 weeks, we observed neither macroscopic signs of colitis, nor elevated total cell numbers in the colonic LP of *Hh*-free DC-LMP1/CD40 animals (Figure 3(e)). In contrast, 14 weeks old *Hh*-pos DC-LMP1/CD40 mice already showed shortened, thickened colon and a strong increase in total colonic cell numbers as consequences of inflammation (Figure 3(e) and ref. ^24^). After 25 weeks, some *Hh*-free DC-LMP1/CD40 mice also showed an inflamed phenotype with a shortened and thickened colon as well as increased cell numbers infiltrating the colon LP (Figure 3(e)). Compared to *Hh*-positive DC-LMP1/CD40 animals, which usually die between 10 and 18 weeks of age,^24^ none of the *Hh*-free transgenic animals showed any signs of morbidity or died before week 25 (data not shown), when they were finally analyzed. Our data show a substantial lack of morbidity and disease of *Hh*-free DC-LMP1/CD40 mice, indicating a crucial role for this microbe in disease initiation and outcome in CD40-mediated colitis.

### SPF-housed DC-LMP1/CD40 mice show no changes in intestinal taxa composition

**Figure 4.**
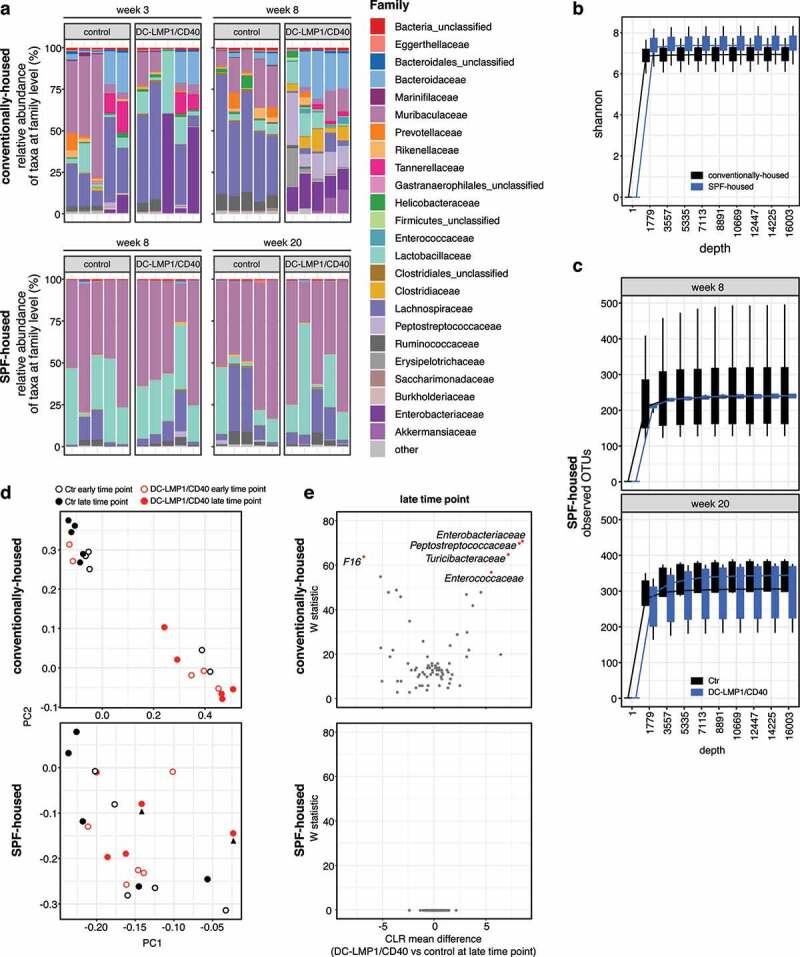
Conventionally housed but not SPF-housed DC-LMP1/CD40 animals show genotype-dependent changes in taxa composition. Analysis of the microbiota in fecal samples from conventionally housed or SPF-housed controls and DC-LMP1/CD40 mice at the indicated time points was based on sequencing the V3-V4 variable regions of the 16S rRNA gene (Illumina MiSeq). Filtered sequences were further processed using Qiime2 version 2020.2. (a) Shown is the relative abundance of taxa at the family level, with each bar representing one animal (n = 5 per group). Taxonomic assignment was performed with classify-sklearn using a classifier trained on SILVA database (Qiime version 132, 99% 16S). (b) Alpha-diversity between conventionally housed and SPF-housed mice shown by Shannon-index. Visualized are boxplots based on values from 10 iterations for each sample at each sampling depth. (c) Number of observed OTUs in 8-week- and 20-week-old SPF-housed Ctr and DC-LMP1/CD40 mice are shown as rarefaction curve. Visualized are boxplots based on values from 10 iterations for each sample at each sampling depth. (d) PCA plots comparing microbial composition in conventionally housed or SPF-housed Ctr and DC-LMP1/CD40 mice at early and late time point. Arrowheads indicate *Hh*-free DC-LMP1/CD40 mice with elevated fecal lipocalin-2 levels as shown in Figure 3(d). (e) At the late time point, differential abundance in 8-week-old conventionally housed or 20-week-old SPF-housed DC-LMP1/CD40 mice vs control littermates was estimated using the ANCOM function after collapsing to taxonomic level 5 and adding pseudo counts. CLR: Centered Log Ratio

To reveal the role of commensals in colitis initiation, we further analyzed intestinal taxa composition in both conventionally housed and SPF-housed mice by 16S rRNA amplicon sequencing of fecal DNA (Figure 4). By analyzing the relative abundance of taxa at family level, SPF-housed animals showed a different taxa composition than conventionally housed animals (Figure 4(a)). However, SPF-housed and conventionally housed mice did not show a difference in alpha-diversity by the Shannon index (Figure 4(b)). We previously observed, that 8-week-old conventionally housed transgenic mice suffering from colitis showed significantly less observed OTU counts when compared to control littermates, indicating dysbiosis upon colitis onset.^24^ However, and in stark contrast, SPF-housed mice did show no reduction in species richness at any time point as revealed by counting OTUs as compared to control littermates (Figure 4(c)).

The principal component analysis (PCA) of conventionally housed (*Hh-*pos) mice revealed that microbial changes are dominated by genotype (Figure 4(d), upper panel). Of note, at the early time point, the two control littermates that cluster with the transgenic mice show some level of *Enterobacteriaceae* (Figure 4(a), upper panel). Conversely, relative abundance of *Enterobacteriaceae* increases only later in the two DC-LMP1/CD40 mice that cluster with the controls at the early time point (Figure 4(a), upper panel). In contrast, there was neither a correlation of PC scores between microbial composition and age nor genotype in animals with an *Hh*-free SPF microbiota (Figure 4(d), lower panel)). Interestingly, there was also no clustering within SPF-housed DC-LMP1/CD40 mice at the late time point (20 weeks), although two of these animals showed high fecal lipocalin-2 levels (Figure 3(d), lower panel), 4(d, lower panel, arrowhead)). The differential taxa abundance at the late time point in conventionally housed (8-week-old) and SPF-housed (20-week-old) mice was further determined using the analysis composition of microbiomes (ANCOM) function (Figure 4(e)). Here, conventionally housed (*Hh-*pos) DC-LMP1/CD40 mice showed *Enterobacteriaceae* blooming, characteristic for dysbiosis during colitis,^42,43^ but also increased abundance of *Peptostreptococcacea*e, *Turicibacteraceae*, and *Enterococcaceae*, while we observed only increased abundance of *F16* in control (*Hh-*pos) littermates (Figure 4(e), upper panel). In contrast, we did not observe significant differential taxa abundances when we compared 20-week-old SPF-housed transgenic and control littermates (Figure 4(e), lower panel). Taken together, *Hh*-free SPF mice do not show genotype-dependent changes in taxa composition, suggesting that the transgenic genetic background of DC-LMP1/CD40 mice is not sufficient to drive development of fatal colitis in the absence of *Hh*.

### DC-LMP1/CD40 mice rapidly develop strong intestinal inflammation upon colonization with Helicobacter hepaticus

**Figure 5.**
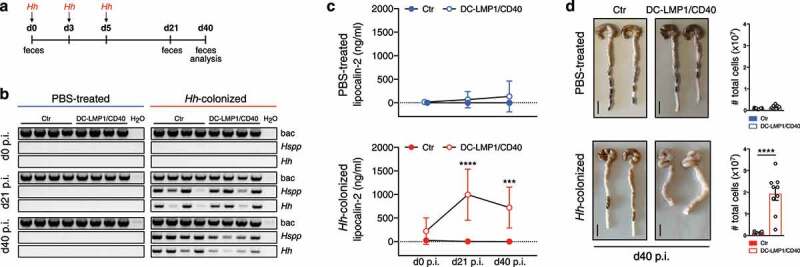
Strong intestinal inflammation upon *Hh*-recolonization. (a) Schematic illustration of colonization of *Hh*-free Ctr and DC-LMP1/CD40 mice with a pure culture of *Hh* by oral gavage at the indicated time points. Feces were collected at the indicated time points and animals were sacrificed 40 days p.i. (b) Bacterial DNA was extracted from fecal samples at the indicated time points from PBS-treated or *Hh*- colonized Ctr and DC-LMP1/CD40 mice at the indicated time points. *Hh*-colonization was confirmed by PCR. Shown is one representative experiment out of two (n = 4). (c) Fecal lipocalin-2 levels in PBS-treated or *Hh*-colonized Ctr and DC-LMP1/CD40 mice were determined by ELISA at the indicated time points. Data is shown as a scatter plot for two pooled experiments with mean ± SEM (n = 9). (d) PBS-treated or *Hh*-colonized Ctr and DC-LMP1/CD40 mice were sacrificed at day 40 p.i. Shown are macroscopic pictures of two representative colons per group (scale bar = 1 cm) as well as bar graphs representing total colon LP cell numbers from two pooled experiments with mean ± SEM (n = 9). bac: bacteria; *Hspp: Helicobacter species; Hh: H. hepaticus.*

To investigate if *Hh* is causal for disease initiation, we inoculated 8-week-old *Hh*-free animals with *Hh* (strain ATCC 51448) by oral gavage (Figure 5(a)). Already at day 21 post-inoculation (p.i.), all DC-LMP1/CD40 mice and control littermates, but not PBS-treated mice were *Hh*-positive as shown by species-specific PCR from feces (Figure 5(b)). On day 40 p.i., when mice were finally sacrificed for analysis, all *Hh*-colonized mice were still *Hh*-positive by PCR (Figure 5(b)). Of note, all mice were negative for the other most relevant *Hspp*, which are also routinely tested according to FELASA recommendations, confirming mono-colonization with *Hh* by oral gavage (Figure S3). Furthermore, *Hh*-infected DC-LMP1/CD40 mice did show significantly elevated fecal lipocalin-2 levels compared to control littermates already on day 21 p.i., indicating a rapid disease onset upon colonization with *Hh* (Figure 5(c)). By d40 p.i., *Hh*-infected DC-LMP1/CD40 mice, but not control littermates showed a strong increase in cells infiltrating the colonic LP as well as a shortened and thickened colon, indicating ongoing inflammation and colitis (Figure 5(d)). Thus, our data reveal that *Hh* is rapidly provoking strong intestinal inflammation in DC-LMP1/CD40 mice, indicating that this bacterial stimulus in the context of CD40-transgene expression is causing the development of early onset colitis.

### Helicobacter hepaticus affects colonic CD4^+^ T cell differentiation

**Figure 6.**
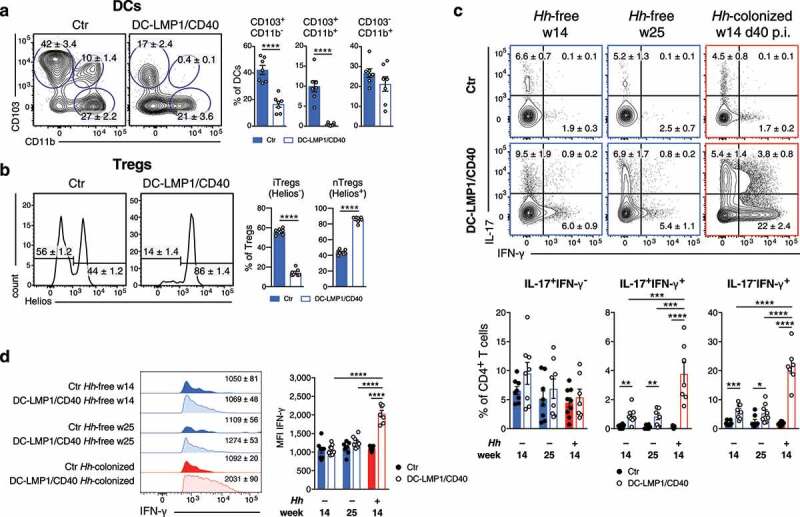
*Hh* affects colonic T cell-fate decisions. Different cell subsets in the colonic LP were analyzed in 14-week-old *Hh*-free Ctr and DC-LMP1/CD40 animals. Shown are representative FACS-plots as well as pooled statistics from two experiments (mean ± SEM, n = 7), illustrating frequencies of the indicated cell subsets. (a) DCs were gated on single, live, CD45^+^, MHCII^+^CD11c^+^ CD64^−^ cells. (b) Tregs were gated on single, live, CD45^+^, CD3^+^CD4^+^ FoxP3^+^CD25^+^, Helios^−^ (iTregs) or Helios^+^ (nTregs). Single-cell suspensions of colonic LP from 14-and 25-week-old *Hh*-free or *Hh*-colonized Ctr or DC-LMP1/CD40 (14-weeks-old, 40 days post *Hh*-colonization) mice were stimulated with PMA/Ionomycin and subsequently stained intracellularly for IL-17 and IFN-γ production at the indicated time points. Bar graphs represent pooled statistics from two experiments (mean ± SEM, n = 7–9) animals. (c) T cells were pre-gated on single, live, CD45^+^, CD3^+^CD4^+^ cells. Shown are representative FACS-plots as well as bar graphs, illustrating the frequencies of the indicated cell subsets within the CD4 T cell population. (d) Shown are representative histograms as well as bar graphs, illustrating the MFI of IFN-γ expression within IFN-γ^+^ CD4^+^ T cells from (c) as median ± SEM. p.i.: post-inoculation

We previously reported the effect of constitutive CD40-signaling on intestinal DCs.^24^ Transgenic animals showed a strong reduction of tolerogenic CD103^+^ DC subsets in the colonic LP and mLNs. As a consequence, iTreg generation was drastically impaired in the large intestine of DC-LMP1/CD40 animals. As the mouse colony used for our previous study was endemically infected by *Hh* (Figure 3(a)), we wondered whether *Hh* is responsible for the phenotypical changes we observed in this colitis model. Therefore, we next analyzed cell subsets in the colonic LP of *Hh*-free DC-LMP1/CD40 and control littermates. We found a strong reduction in CD103^+^CD11b ^–^ as well as CD103^+^CD11b^+^ intestinal DCs in 14-week-old *Hh*-free DC-LMP1/CD40 animals but not control littermates (Figure 6(a)), similar to what we previously described for *Hh*-pos animals.^24^ Furthermore, Helios ^–^ iTregs were significantly reduced in the colonic LP of 14-week-old *Hh*-free DC-LMP1/CD40 mice, but not control animals (Figure 6(b)), as published previously for *Hh*-pos animals.^24^ Of note, the reduction of intestinal CD103^+^ DCs as well as iTregs was also found in 25-week-old *Hh*-free DC-LMP1/CD40 mice, but not control animals (Figure S4). Therefore, we conclude that the phenotypical changes in DC-LMP1/CD40 mice are rather a consequence of the transgene expression in DCs, suggesting that *Hh* has no direct impact on DC or Treg differentiation in this model.

We also know from our previous study that DC-LMP1/CD40 mice show a strong increase in IL-17^+^IFN-γ^+^ Th17/Th1 and IFN-γ^+^ Th1 cells in the colonic LP.^24^ To evaluate the role of *Hh* in this CD4^+^ T cell differentiation process, we compared IL-17- and IFN-γ-producing CD4^+^ T cells in different mice (Figure 6(c)). We did not detect significantly different frequencies of IL-17^+^CD4^+^ T cells in DC-LMP1/CD40 or control animals, neither in *Hh*-free nor in *Hh*-colonized animals (Figure 6(c)). However, at day 40 p.i., *Hh*-positive DC-LMP1/CD40 mice had significantly increased frequencies of both IL-17^+^IFN-γ^+^ Th17/Th1 and IFN-γ^+^ Th1 cells when compared to 14- or 25-week-old *Hh*-free DC-LMP1/CD40 mice (Figure 6(c)). Of note, also *Hh*-free transgenic animals showed some induction of IL-17^+^IFN-γ^+^ Th17/Th1 and IFN-γ^+^ Th1 cells in the colonic LP when compared to appropriate control littermates (Figure 6(c)). However, when we analyzed the mean fluorescence intensity (MFI) of IFN-g expression in IFN-γ^+^ CD4^+^ T cells, only Th1 cells from *Hh*-positive DC-LMP1/CD40 mice produced significantly higher amounts of IFN-γ when compared to *Hh*-free transgenic animals (Figure 6(d)). Taken together, our results suggest that *Hh* has the potential to rapidly initiate the transdifferentiation of nonpathogenic Th17 into pathogenic Th1 cells in the colonic LP devoid of tolerogenic CD103^+^ DCs and iTregs.^25^ However, this may not be an exclusive property of *Hh* but is also induced in *Hh*-free mice by other commensals or mechanisms, yet with much less efficacy.

## Discussion

In this study, we identified the murine commensal *Helicobacter hepaticus* as a driver of pathogenesis in a CD40-mediated model of colitis. Upon very early colitis onset, DC-LMP1/CD40 animals showed elevated serum IgG- as well as IgA-levels. Although IgA is mainly produced locally in the gut, we observed elevated levels of IgA and increased anti-commensal IgA also in sera of mice. To ensure mucosal homeostasis, the gut sustains tolerance toward commensals by restraining bacteria by various mechanisms, including the secretion of protective anti-microbial peptides and bacteria-specific IgA. Thus, bacteria-specific antibodies are not detectable in sera from healthy SPF-housed mice.^44^ It is interesting to note that IgA-coating seems to occur preferentially with microbiota which have enhanced potential for inflammation (reviewed in ref.^45^). Furthermore, previous reports showed that IgA increased the invasiveness of segmented filamentous bacteria^46^ and *Helicobacter* species.^47^ As microbiota were strongly coated with IgA in DC-LMP1/CD40 mice,^24^ elevated IgA could contribute to colitis, according to previous studies. However, during inflammatory conditions, systemic antibodies are produced as a consequence of mucosal barrier dysfunction and thus increased exposure of commensals at systemic sites.^48^ The presence of commensal-specific serum antibodies in DC-LMP1/CD40 mice therefore suggests that compartmentalization might be broken in mice with colitis, leading to systemic antibody responses.

Only recently, it was reported that especially members of Proteobacteria are able to induce T cell-dependent serum IgA responses in conventionally housed mice to protect them from lethal sepsis.^49^ In this study, commensal *Helicobacter muridarum* was identified as the driving species, which would induce mucosal IgA-secreting plasma cells as well as IgA^+^ bone marrow plasma cells.^49^ Our data suggest that dysbiosis in DC-LMP1/CD40 mice affects the dissemination of bacteria, inducing systemic IgG as well as IgA production. This hypothesis is also supported by human studies, reporting elevated serum antibody levels in IBD patients.^50,51^ However, we also found certain levels of bacteria-specific serum IgG in control littermates. One explanation for this observation might be that we used conventionally but not SPF-housed mice for a part of our study. This seems more analogous to healthy humans, where also some level of systemic bacteria-specific IgG exists, which does increase further during IBD.^48^

We identified serum antibodies from transgenic DC-LMP1/CD40 animals being bacteria-specific and recognizing the 60 kDa GroEL, a protein that is also secreted by *Helicobacter*.^52^ The fact that only the 60 kDa chaperonin from *Hh* was identified with this method was surprising, but heat shock proteins have been reported as immunodominant antigens, inducing humoral and cellular immune responses during several diseases in humans and mice. For instance, αHsp60 antibodies are found in patients with tuberculosis and in mice infected with *Mycobacterium tuberculosis*.^53,54^ Pathogen-derived 60 kDa chaperonin induces pro-inflammatory cytokines *in vitro*^55^ and mice infected with *Yersinia enterocolitica* produce 60 kDa chaperonin-specific T cells involved in anti-pathogenic immune response.^53^ Serum antibodies specific for *H. pylori* Hsp60 were also reported in patients with gastric cancer.^56^ Our data suggest that *Hh* is involved in disease development and that the *Hh* 60 kDa chaperonin is an immunodominant antigen in the CD40-mediated colitis model.

*Hh* is known as an endemic pathobiont in many mouse colonies^12,13^ and elicits intestinal inflammation in immunodeficient or immunocompromised mice. This mimics human IBD as shown in several mouse models where *Hh* elicits spontaneous colitis.^16–18,20,57^ Although all of our conventionally housed mice were positive for the *Helicobacter* genus, only control mice remained consistently positive for *Hh*. In contrast, young DC-LMP1/CD40 mice showed already reduced prevalence, while *Hh* was hardly detectable in older DC-LMP1/CD40 mice. One reason for this phenomenon might be the clearance of *Hh*, eventually because of increased anti-*Hh* serum IgG and IgA levels. Alternatively, *Hh* may be simply displaced, for example, by *Enterobacteriaceae*, which bloom^42,43^ during inflammation in DC-LMP1/CD40 animals. This suggests that *Hh* is causing disease initiation but eventually not its maintenance and progression.

In contrast, *Hh*-free DC-LMP1/CD40 mice only developed mild intestinal inflammation at the age of 5 to 6 months, when *Hh*-positive transgenic animals had already died of the disease. Interestingly, also IL-10-deficient mice developed intestinal inflammation with delayed onset and less severity in 5- to 6-month-old animals when maintained under SPF conditions.^18^ In addition, *Hh*-free transgenic mice with Treg-specific c-Maf deficiency developed mild spontaneous colitis at a later age of 6 to 12 months.^57^ The protection from early disease onset in *Hh*-free DC-LMP1/CD40 mice suggests that *Hh* might be a very potent disease driver. Although we could not find any evidence for specific commensals involved in disease initiation in aged *Hh*-free SPF-housed transgenic mice, also other bacteria may cause the disease, although much weaker, with later onset and in fewer mice. When kept under SPF-conditions, the lack of iTregs and CD103^+^ DCs in DC-LMP1/CD40 mice had no impact on the taxa composition of the intestinal microbiota of these mice. Therefore, we assume that the dramatic changes in taxa composition observed in the same mice under conventional housing were due to inflammation caused or initiated by *Hh*.

While we determined *Hh* as a disease driver in CD40-mediated colitis model, this microbe did not have a direct impact, neither on DC nor on Treg differentiation in the colon LP, as CD103^+^ DCs and iTregs were similarly reduced in both, *Hh*-free and *Hh*-positive DC-LMP1/CD40 animals.^24^ In contrast, *Hh* affected CD4^+^ effector T cell differentiation in the colon, which significantly increased IL-17^+^IFN-γ^+^ Th17/Th1 and IFN-γ^+^ Th1 cells. Moreover, in IL-10^−/−^ mice, *Hh*-infection induced pathogenic *Hh*-specific IL-17^+^IFN-γ^+^ Th17/Th1 cells,^58^ probably because of the inability of Tregs to restrain colitogenic Th17 cells in *Hh*-positive IL-10^−/−^ mice.^57^

Our data revealed that the intestinal microbiota can modulate the host immune response with an impact on disease onset, progression, and severity. Here, we identified *Hh* as a disease driver in the DC-LMP1/CD40 colitis model. In the context of constitutive CD40-signaling in DCs, we could show that Hh causes an increase in IFN-γ expression in Th17 cells which correlates with tissue damage in the intestine. This may result from transdifferentiation of nonpathogenic Th17 cells into pathogenic Th1 cells. However, this hypothesis has to be confirmed by fate mapping experiments, using the IL-17A-Cre mice and an eYFP reporter in the Rosa26-locus. Yet, this is challenging as the transgene LMP1/CD40 is also integrated into the Rosa26-locus.

Our results are also of relevance for other studies using conventionally housed mice as *Hh* is endemic in many mouse colonies. Our data further confirm the important role of the gut microbial composition during health and disease and reveal that a single bacterial species dramatically affects host immunity, if compromised. The identification of other potentially disease driving bacteria and specific bacterial antigens and underlying mechanisms in IBD is important. This further contributes to understanding the complex interaction of microbiota and host immune cells for development and improvement of personalized therapeutic strategies in IBD.

## Materials and methods

### Mice

DC-LMP1/CD40 mice were generated as previously described.^24^ Briefly, CD11cCre mice^34^ were crossed with LMP1/CD40^fl/flSTOP35^ animals to obtain DC-LMP1/CD40 mice with constitutive CD11c-specific CD40-signaling.

Mice were analyzed in sex- and age-matched groups of 8–25 weeks of age, unless otherwise stated. Littermate animals were used as controls in a non-randomized, non-blinded fashion. Animal experiment permissions were granted by the animal ethics committee Regierung von Oberbayern, Munich, Germany (55.2.1.54–2532-22-2017). Mice were bred and maintained under conventional conditions at the animal facility of the Institute for Immunology, Ludwig-Maximilians-Universität München. After embryo transfer rederivation performed by ENVIGO (Huntingdon, United Kingdom), all mice were kept under specified pathogen-free conditions (tested quarterly according to FELASA-14 recommendations) and housed in groups of 2–3 animals in IVCs (Tecniplast, Germany) at 12 h/12 h light/dark cycle. Mice had free access to water (acidified and desalinated) and standard rodent chow (Altromin, 1310 M).

### Single-cell preparation

Single-cell suspensions of lymph nodes were prepared by mashing organs through a 100 μM cell strainer. Samples were washed with PBS and stored on ice for further analysis. The number of living cells was determined using the CASY Counter (OMNI Life Science). Cells from the colonic LP were isolated as previously described.^24^ Briefly, the colon was removed, cleaned from fecal content, opened longitudinally, cut into pieces and predigested in Hank’s balanced salt solution (HBSS) supplemented with 10 mM HEPES and 10 mM EDTA for 10 min on a shaker at 37°C. Pieces were further digested for 30 min and then twice for 20 min with a mixture of Collagenase IV (157 Wuensch units ml^−1^, Worthington), DNAse I (0.2 mg ml^−1^ dissolved in PBS) and Liberase (0.65 Wuensch units ml^−1^, both Roche, dissolved in HBSS supplemented with 8% FCS). Lymphocytes were purified with a 40/80 Percoll gradient and the number of living cells was determined using the CASY Counter.

### Flow cytometry analysis

Where possible, 2 × 10^6^ cells were stained with titred antibodies in PBS containing 2% FCS and 0.01% NaN_3_ (FACS buffer) for 20 min at 4°C in the dark. Cells were washed once and used for direct acquisition on BD FACSCanto or fixed using 2% paraformaldehyde in FACS buffer and measured the next day. Dead cells were excluded using Zombie Aqua Fixable Viability Kit (BioLegend, Cat: 423102). For intracellular cytokine stainings, cells were fixed and permeabilized for 30 min at 4°C in the dark after extracellular staining using BD Cytofix/Cytoperm (Fixation and Permeabilization Solution, BD Biosciences, Cat: 51–2090KZ) according to manufacturer’s instruction. Cells were washed and stained with the indicated antibodies in 50 μl BD Perm/Wash Buffer (BD Biosciences, Cat: 51–2091KZ) for 30 min at 4°C in the dark. For transcription factor staining, cells were fixed and permeabilized after extracellular staining in 1x Fixation/Permeabilization solution (eBioscience, Cat: 00–5523-00) for 30 min at 4°C in the dark according to the manufacturer’s instructions. Cells were washed twice with 1x Permeabilization Buffer (eBioscience, Cat: 00–5523-00) and stained with the indicated antibodies in 50 μl 1x Permeabilization Buffer for 30 min at 4°C in the dark. Afterward, cells were washed once and acquired on BD FACSCanto.

The following antibodies were used: FoxP3 (FJK-16s; eFlour660, dil. 1:50), Helios (22F6; FITC, dil. 1:400) (eBioscience); CD25 (PC61; PerCP, dil. 1:400), CD103 (M290; PE, dil. 1:150) (BD Pharmingen); CD11b (M1/70; APC-eFluor780, dil. 1:400) (Invitrogen); CD3 (17A2; AlexaFluor488, dil. 1:400; Pe-Cy7, dil. 1:400), CD4 (RM4-5; PerCP, 1:800; GK1.5; APC-Cy7, dil. 1:400), CD11c (N418; Pe-Cy7, dil. 1:400), CD45 (30-F11; BV421, dil. 1:400), CD64 (X54-5/7.1; APC, dil. 1:200), IL-17A (TC11-18H10.1; PE, dil. 1:200), IFN-γ (XMG1.2; APC, dil. 1:400), MHC class II (I-A/I-E) (M5/114.15.2; FITC, PerCP, dil. 1:800) (BioLegend). Data analysis was performed using FlowJo version 10 (TreeStar, Ashland, OR, USA).

### Ex vivo T cell restimulation

2 × 10^6^ cells were stimulated for 4 h at 23°C with 40 ng ml^−1^ PMA and 1 μg ml^−1^ ionomycin in the presence of 2 μM Monensin (Golgi-Stop, BD Biosciences, Cat: 51–2092KZ). Cells were washed twice with FACS buffer and stained for extracellular markers, fixed/permeabilized and stained for intracellular markers as described above.

### ELISA for fecal Lipocalin-2

Fecal samples were reconstituted in PBS containing 0.1% Tween 20 (100 mg ml^−1^) and vortexed for 20 min for homogenization. Upon centrifugation for 15 min at 100 x g at 4°C, supernatants were centrifuged again for 10 min at 10,000 x g at 4°C. Supernatants were analyzed for lipocalin-2 content using Quantikine ELISA kit for mouse Lipocalin-2/NGAL (R&D Systems, Cat: MLCN20).

### Determination of serum antibody concentrations

Blood from mice was collected by terminal cardiac puncture and transferred into a Microtainer tube (BD Biosciences, Cat: 365963). After incubation at room temperature for at least 3 h, the coagulated blood was centrifuged at 8000 rpm for 5 min at 21°C and serum was frozen at −20°C until use. Serum antibody concentrations were determined using Mouse IgG total Ready-SET-Go! or Mouse IgA Ready-SET-Go! ELISA (eBioscience, Cat: 88–50400 and 88–50450), according to manufacturer’s instructions.

### Immunoprecipitation of bacterial antigens

Cecal bacterial lysate (CBL) was prepared as previously described.^24^ Briefly, pooled cecal content from C57BL/6 mice was homogenized, spun down and protein concentration within the supernatant was determined. Identification of bacterial antigens within the CBL was performed by using serum antibodies from control and DC-LMP1/CD40 mice for immunoprecipitation followed by Mass Spectrometry. Therefore, 50 µl protein G beads (Dynabeads Protein G, Invitrogen, Cat: 10004D) were coupled with 2.5 µg serum IgG from Ctr or DC-LMP1/CD40 mice for 10 min at room temperature. 1600 µg CBL was added to the coated beads for 30 min at room temperature and the complex was washed three times with PBS/Tween 0.02% followed by an additional 3 rounds of washing with 50 mM NH_4_HCO_3_. Samples were stored at −20°C until LC-MS/MS was performed by the Protein Analysis Unit (Biomedical Center, LMU Munich).

### On-beads trypsin digest and Mass Spectrometry

Following the immunoprecipitation procedure described above, beads were incubated with 100 µl of 10 ng µl^−1^ trypsin solution in 1 M Urea and 50 mM NH_4_HCO_3_ for 30 min at 25°C for trypsin digestion. The supernatant was collected, beads washed twice with 50 mM NH_4_HCO_3_, and all three supernatants collected together and incubated overnight at 25°C at 800 rpm after addition of dithiothreitol to 1 mM. Iodoacetamide was added at a final concentration of 27 mM and the samples were incubated at 25°C for 30 min in the dark. 1 µl of 1 M dithiothreitol was added to the samples and incubated for 10 min to quench the iodoacetamide. Finally, 2.5 µl of trifluoroacetic acid was added and the samples were subsequently desalted using C18 Stage tips. Samples were evaporated to dryness, resuspended in 15 µl of 0.1% formic acid solution, and injected into an Ultimate 3000 RSLCnano system (Thermo), separated by a 15-cm analytical column (75 μm ID home-packed with ReproSil-Pur C18-AQ 2.4 μm from Dr. Maisch) with a 50 min gradient from 5% to 60% acetonitrile in 0.1% formic acid. The effluent from the HPLC was directly electrosprayed into a Qexactive HF (Thermo) operated in data-dependent mode to automatically switch between full-scan MS and MS/MS acquisition. Survey full-scan MS spectra (from m/z 375–1600) were acquired with resolution R = 60,000 at m/z 400 (AGC target of 3 × 10^6^). The 10 most intense peptide ions with charge states between 2 and 5 were sequentially isolated to a target value of 1 × 10^5^ and fragmented at 27% normalized collision energy. Typical mass spectrometric conditions were: spray voltage, 1.5 kV; no sheath and auxiliary gas flow; heated capillary temperature, 250°C; ion selection threshold, 33,000 counts. MaxQuant 1.5.2.8 was used to identify proteins and quantify by intensity-based absolute quantification (iBAQ) with the following parameters: Database, uniprot_proteomes_Bacteria_151113.fasta; MS tol, 10 ppm; MS/MS tol, 10 ppm; Peptide FDR, 0.1; Protein FDR, 0.01 Min. peptide Length, 5; Variable modifications, Oxidation (M); Fixed modifications, Carbamidomethyl (C); Peptides for protein quantitation, razor and unique; Min. peptides, 1; Min. ratio count, 2. Identified proteins were considered as interaction partners if their MaxQuant iBAQ values were greater than log2 twofold enrichment and *p*-value 0.05 (ANOVA) when compared to the control.

### Culture and lysate preparation of Hh

The *Helicobacter hepaticus* strain *Hh*-2 (ATCC 51448)^59^ was purchased from the Leibniz Institute DSMZ – German Collection of Microorganisms and Cell Cultures (DSM No.22909) and cultivated at the Max von Pettenkofer-Institute, LMU Munich. Bacteria from cryo stock were resuspended in Brain Heart Infusion (BHI) medium and put on blood agar plates (Columbia agar with 5% sheep blood, BD, Cat: 4354005). Plates were incubated in a chamber with anaerobic conditions (83% N_2_, 10% CO_2_, 7% H_2_) for 4 days at 37°C. A subculture was cultivated further on in BHI medium with 3% sheep serum in a culture flask in the chamber with anaerobic conditions for additional 4 days at 37°C.

For *Hh* lysate (*Hh*L) preparation, bacterial cells were harvested and washed 2–3 times with PBS. Cell pellets were resuspended in PBS and lyzed by sonification with the Sonifier 150 Cell Disruptor (Branson) 6 times for 3 min at level 3 on ice. Lyzed cells were centrifuged at 20,000 x g for 30 min at 4°C and the supernatant was mixed with a protease inhibitor (cOmplete ULTRA Tablets, Roche, Sigma-Aldrich, Cat: 05892953001). Protein concentration was determined using the Qubit Protein Assay Kit and Fluorometer (Invitrogen), according to the manufacturer’s instructions and the lysate was stored at −20°C until use for immunoblot or ELISA.

### ELISA for commensal- or Hh-specific antibodies

This assay was performed as previously described^24^ with the following modifications. The CBL was diluted in carbonate buffer to a final concentration of 1 µg ml^−1^. *Hh*L was prepared as described above and diluted in carbonate buffer to a final concentration of 0.1 µg ml^−1^. Differences in serum antibody concentrations between Ctr and DC-LMP1/CD40 mice were adjusted by using 2.5 µg ml^−1^ serum IgG or 6.5 µg ml^−1^ serum IgA for all samples.

### Immunoblotting for commensal- or Hh-specific antibodies

Serum IgG or IgA reactivity toward CBL or *Hh*L was analyzed by immunoblot analysis. For screening multiple sera from Ctr and DC-LMP1/CD40 animals, the Mini-PROTEAN II Multiscreen Apparatus (Bio-Rad, Cat: 1704017) was used. CBL (30 µg per lane or 600 µg for Mini-PROTEAN II Multiscreen Apparatus) or *Hh*L (20 µg per lane or 200 µg for Mini-PROTEAN II Multiscreen Apparatus) were separated by SDS-PAGE and transferred to a nitrocellulose membrane. Sera of mice were used as primary antibodies. Differences in serum antibody concentrations between Ctr and DC-LMP1/CD40 mice were adjusted by using 2.5 µg ml^−1^ serum IgG or 1 µg ml^−1^ serum IgA for all samples. In some experiments, mouse IgG1 anti-human heat shock protein 60 (aHSP60) antibody (clone LK-2, Enzo, Cat: ADI-SPA-807-E) was additionally used as the primary antibody (1:10,000 in PBS/1% nonfat dried milk). HRP-conjugated secondary antibodies were used as follows: goat anti-mouse IgG-HRP (SouthernBiotech, Cat: 1030–05; 1:10,000) or goat anti-mouse IgA-HRP (SouthernBiotech, Cat: 1040–05; 1:10,000). Western Lightning Plus-ECL Detection Reagent (PerkinElmer, Cat: NEL103E001EA) and X-ray films (Amersham, Cat: 45–001-504) were used for protein detection.

### Bacteria screening PCR

Mice were screened for bacterial colonization by PCR using the 16S rRNA gene as target. Genomic DNA was isolated from fecal pellets with the QIAamp Fast DNA Stool Mini Kit (Qiagen), according to the manufacturer’s instructions. 5–10 ng DNA was used for amplification with MyTaq Polymerase (Bioline). The PCR cycling conditions were as follows: denaturation at 94°C for 1 min, annealing for bacteria at 58°C, for *Hspp* and *Hh* at 61°C and for *Ht, Hr* and *Hb* at 55°C for 1 min, elongation at 72°C for 1 min (35 cycles) and final elongation at 72°C for 7 min.

The following primer sets were used: bacteria (forward primer: 5ʹ-TCCTACGGGAGGCAGCAGT-3ʹ, reverse primer: 5ʹ-GGACTACCAGGGTATCTAATCCTGTT-3ʹ, 467 bp);^60^ *Hspp* (forward primer: 5ʹ-TATGACGGGTATCCGGC-3ʹ, reverse primer: 5ʹ-ATTCCACCTACCTCTCCCA-3ʹ, 375 bp);^61^ *Hh* (forward primer: 5ʹ-GCATTTGAAACTGTTACTCTG-3ʹ, reverse primer: 5ʹ-CTGTTTTCAAGCTCC- CC-3ʹ, 417 bp);^12^ *Ht* (forward primer: 5ʹ-TTAAA-GATATTCTAGGGGTATAT-3ʹ, reverse primer: 5ʹ-TCTCCCATCTCTAGAGTGA-3ʹ, 455 bp);^62^ *Hr* (forward primer: 5ʹ-GTCCTTAGTTGCTAACTATT-3ʹ, reverse primer: 5ʹ- AGATTTGCTCCATTTCACAA-3ʹ, 166 bp);^63^ *Hb* (forward primer: 5ʹ-AGAACTGCATTTGAAACTACTTT-3ʹ, reverse primer: 5ʹ- GGTATTGCATCTCTTTGTATGT-3ʹ, 638 bp).^64^

### 16S rRNA gene amplicon sequencing and taxonomic profiling

Microbiome analysis was done on whole DNA extracted from mouse fecal samples and is based on sequencing the V3-V4 variable regions of the 16S rRNA gene as previously described.^24^ Amplicons were analyzed with mothur v. 1.43.0.^65^ to remove chimeric sequences with the “chimera.vsearch”-command (default settings). Sequences were further processed using Qiime2 version 2020.2, Taxonomic assignment was performed with classify sklearn using a classifier trained on SILVA database (Qiime version 132, 99% 16S). Differential abundance was estimated using the ANCOM function^66^ after collapsing to taxonomic level five and adding pseudo counts.

### Colonization with Hh by oral gavage

Bacterial suspensions cultured as described above were used for oral inoculation to mice. *Hh* identity was confirmed by 16S RNA gene sequencing. Bacterial density was determined by OD measurements at 600 nm. Appropriate amount of suspension was washed with PBS and then adjusted to OD (600) = 3.0. 8-week-old mice were kept under specific and opportunistic pathogen-free conditions for the time of the experiment and inoculated with 100 µl of the suspension by oral gavage at day 0, 3 and 5, for a total of 3 doses. Animals were analyzed 40 days post-inoculation.

### Statistics

For absolute cell numbers, the percentage of living cells of a certain subset was multiplied by the number of living cells as determined by CASY Counter. Unless otherwise stated, significance was determined using unpaired Student’s *t*-test and defined as follows: *P < .05, **P < .01, and ***P < .001 and ****P < .0001. Error bars represent mean ± SEM.

## Supplementary Material

Supplemental Material

## Data Availability

The mass spectrometry proteomics data have been deposited to the ProteomeXchange Consortium via the PRIDE (http://www.proteomexchange.org) partner repository with the dataset identifier PXD018025. 16S rRNA amplicon sequencing data have been deposited in the NCBI Sequence Read Archive under Accession Number SRX1799186 and PRJNA631278.

## References

[1] Sekirov I, Russell SL, Antunes LC, Finlay BB. Gut microbiota in health and disease. Physiol Rev. 2010;90:859–20. doi:10.1152/physrev.00045.2009. 20664075

[2] Kamada N, Seo SU, Chen GY, Nunez G. Role of the gut microbiota in immunity and inflammatory disease. Nat Rev Immunol. 2013;13:321–335. doi:10.1038/nri3430. 23618829

[3] Littman DR, Pamer EG. Role of the commensal microbiota in normal and pathogenic host immune responses. Cell Host Microbe. 2011;10:311–323. doi:10.1016/j.chom.2011.10.004. 22018232 PMC3202012

[4] Maynard CL, Elson CO, Hatton RD, Weaver CT. Reciprocal interactions of the intestinal microbiota and immune system. Nature. 2012;489:231–241. doi:10.1038/nature11551. 22972296 PMC4492337

[5] Takahashi K, Nishida A, Fujimoto T, Fujii M, Shioya M, Imaeda H, Inatomi O, Bamba S, Sugimoto M, Andoh A. Reduced abundance of butyrate-producing bacteria species in the fecal microbial community in Crohn’s disease. Digestion. 2016;93:59–65. doi:10.1159/000441768. 26789999

[6] Seksik P, Rigottier-Gois L, Gramet G, Sutren M, Pochart P, Marteau P, Jian R, Dore J. Alterations of the dominant faecal bacterial groups in patients with Crohn’s disease of the colon. Gut. 2003;52:237–242. doi:10.1136/gut.52.2.237. 12524406 PMC1774977

[7] Manichanh C, Rigottier-Gois L, Bonnaud E, Gloux K, Pelletier E, Frangeul L, Nalin R, Jarrin C, Chardon P, Marteau P, et al. Reduced diversity of faecal microbiota in Crohn’s disease revealed by a metagenomic approach. Gut. 2006;55:205–211. doi:10.1136/gut.2005.073817. 16188921 PMC1856500

[8] Ni J, Wu GD, Albenberg L, Tomov VT. Gut microbiota and IBD: causation or correlation? Nat Rev Gastroenterol Hepatol. 2017;14:573–584. doi:10.1038/nrgastro.2017.88. 28743984 PMC5880536

[9] Ivanov II, Atarashi K, Manel N, Brodie EL, Shima T, Karaoz U, Wei D, Goldfarb KC, Santee CA, Lynch SV, et al. Induction of intestinal Th17 cells by segmented filamentous bacteria. Cell. 2009;139:485–498. doi:10.1016/j.cell.2009.09.033. 19836068 PMC2796826

[10] Ivanov II, Frutos Rde L, Manel N, Yoshinaga K, Rifkin DB, Sartor RB, Finlay BB, Littman DR. Specific microbiota direct the differentiation of IL-17-producing T-helper cells in the mucosa of the small intestine. Cell Host Microbe. 2008;4:337–349. doi:10.1016/j.chom.2008.09.009. 18854238 PMC2597589

[11] Atarashi K, Tanoue T, Shima T, Imaoka A, Kuwahara T, Momose Y, Cheng G, Yamasaki S, Saito T, Ohba Y, et al. Induction of colonic regulatory T cells by indigenous Clostridium species. Science. 2011;331:337–341. doi:10.1126/science.1198469. 21205640 PMC3969237

[12] Shames B, Fox JG, Dewhirst F, Yan L, Shen Z, Taylor NS. Identification of widespread Helicobacter hepaticus infection in feces in commercial mouse colonies by culture and PCR assay. J Clin Microbiol. 1995;33:2968–2972. https://www.ncbi.nlm.nih.gov/pubmed/8576355 8576355 10.1128/jcm.33.11.2968-2972.1995 PMC228616

[13] Taylor NS, Xu S, Nambiar P, Dewhirst FE, Fox JG. Enterohepatic Helicobacter species are prevalent in mice from commercial and academic institutions in Asia, Europe, and North America. J Clin Microbiol. 2007;45:2166–2172. doi:10.1128/JCM.00137-07. 17507523 PMC1933014

[14] Fox JG, Li X, Yan L, Cahill RJ, Hurley R, Lewis R, Murphy JC. Chronic proliferative hepatitis in A/JCr mice associated with persistent Helicobacter hepaticus infection: a model of helicobacter-induced carcinogenesis. Infect Immun. 1996;64:1548–1558. doi:10.1128/IAI.64.5.1548-1558.1996 8613359 PMC173960

[15] Ihrig M, Schrenzel MD, Fox JG. Differential susceptibility to hepatic inflammation and proliferation in AXB recombinant inbred mice chronically infected with Helicobacter hepaticus. Am J Pathol. 1999;155:571–582. doi:10.1016/S0002-9440(10)65152-8. 10433949 PMC1868606

[16] Cahill RJ, Foltz CJ, Fox JG, Dangler CA, Powrie F, Schauer DB. Inflammatory bowel disease: an immunity-mediated condition triggered by bacterial infection with Helicobacter hepaticus. Infect Immun. 1997;65:3126–3131. doi:10.1128/IAI.65.8.3126-3131.1997 9234764 PMC175441

[17] Erdman SE, Poutahidis T, Tomczak M, Rogers AB, Cormier K, Plank B, Horwitz BH, Fox JG. CD4+ CD25+ regulatory T lymphocytes inhibit microbially induced colon cancer in Rag2-deficient mice. Am J Pathol. 2003;162:691–702. doi:10.1016/S0002-9440(10)63863-1. 12547727 PMC1851156

[18] Kullberg MC, Ward JM, Gorelick PL, Caspar P, Hieny S, Cheever A, Jankovic D, Sher A. Helicobacter hepaticus triggers colitis in specific-pathogen-free interleukin-10 (IL-10)-deficient mice through an IL-12- and gamma interferon-dependent mechanism. Infect Immun. 1998;66:5157–5166. doi:10.1128/IAI.66.11.5157-5166.1998. 9784517 PMC108643

[19] Chin EY, Dangler CA, Fox JG, Schauer DB. Helicobacter hepaticus infection triggers inflammatory bowel disease in T cell receptor alphabeta mutant mice. Comp Med. 2000;50:586–594. https://www.ncbi.nlm.nih.gov/pubmed/11200563. 11200563

[20] Burich A, Hershberg R, Waggie K, Zeng W, Brabb T, Westrich G, Viney JL, Maggio-Price L. Helicobacter-induced inflammatory bowel disease in IL-10- and T cell-deficient mice. Am J Physiol Gastrointest Liver Physiol. 2001;281:G764–778. doi:10.1152/ajpgi.2001.281.3.G764. 11518689

[21] Bachmaier K, Penninger JM. Chlamydia and antigenic mimicry. Curr Top Microbiol Immunol. 2005;296:153–163. doi:10.1007/3-540-30791-5_9. 16323424

[22] Fust G, Uray K, Bene L, Hudecz F, Karadi I, Prohaszka Z. Comparison of epitope specificity of anti-heat shock protein 60/65 IgG type antibodies in the sera of healthy subjects, patients with coronary heart disease and inflammatory bowel disease. Cell Stress Chaperones. 2012;17:215–227. doi:10.1007/s12192-011-0301-7. 22038196 PMC3273563

[23] Cappello F, Mazzola M, Jurjus A, Zeenny MN, Jurjus R, Carini F, Leone A, Bonaventura G, Tomasello G, Bucchieri F, et al. Hsp60 as a novel target in IBD management: a prospect. Front Pharmacol. 2019;10:26. doi:10.3389/fphar.2019.00026. 30800066 PMC6376446

[24] Barthels C, Ogrinc A, Steyer V, Meier S, Simon F, Wimmer M, Blutke A, Straub T, Zimber-Strobl U, Lutgens E, et al. CD40-signalling abrogates induction of RORgammat(+) Treg cells by intestinal CD103(+) DCs and causes fatal colitis. Nat Commun. 2017;8:14715. doi:10.1038/ncomms14715. 28276457 PMC5347138

[25] Ogrinc Wagner A, Friedrich V, Barthels C, Marconi P, Blutke A, Brombacher F, Brocker T. Strain specific maturation of Dendritic cells and production of IL-1beta controls CD40-driven colitis. PLoS One. 2019;14:e0210998. doi:10.1371/journal.pone.0210998. 30653608 PMC6336277

[26] Kusters P, Seijkens T, Burger C, Legein B, Winkels H, Gijbels M, Barthels C, Bennett R, Beckers L, Atzler D, et al. Constitutive CD40 signaling in dendritic cells limits atherosclerosis by provoking inflammatory bowel disease and ensuing cholesterol malabsorption. Am J Pathol. 2017;187:2912–2919. doi:10.1016/j.ajpath.2017.08.016. 28935569

[27] Gonsky R, Deem RL, Landers CJ, Haritunians T, Yang S, Targan SR. IFNG rs1861494 polymorphism is associated with IBD disease severity and functional changes in both IFNG methylation and protein secretion. Inflamm Bowel Dis. 2014;20:1794–1801. doi:10.1097/MIB.0000000000000172. 25171510 PMC4327845

[28] Danese S, Katz JA, Saibeni S, Papa A, Gasbarrini A, Vecchi M, Fiocchi C. Activated platelets are the source of elevated levels of soluble CD40 ligand in the circulation of inflammatory bowel disease patients. Gut. 2003;52:1435–1441. doi:10.1136/gut.52.10.1435. 12970136 PMC1773814

[29] Liu Z, Colpaert S, D’Haens GR, Kasran A, de Boer M, Rutgeerts P, Geboes K, Ceuppens JL. Hyperexpression of CD40 ligand (CD154) in inflammatory bowel disease and its contribution to pathogenic cytokine production. J Immunol. 1999;163:4049–4057. https://www.ncbi.nlm.nih.gov/pubmed/10491009. 10491009

[30] Ludwiczek O, Kaser A, Tilg H. Plasma levels of soluble CD40 ligand are elevated in inflammatory bowel diseases. Int J Colorectal Dis. 2003;18:142–147. doi:10.1007/s00384-002-0425-4. 12548417

[31] Danese S, Sans M, Fiocchi C. The CD40/CD40L costimulatory pathway in inflammatory bowel disease. Gut. 2004;53:1035–1043. doi:10.1136/gut.2003.026278. 15194658 PMC1774101

[32] Hart AL, Al-Hassi HO, Rigby RJ, Bell SJ, Emmanuel AV, Knight SC, Kamm MA, Stagg AJ. Characteristics of intestinal dendritic cells in inflammatory bowel diseases. Gastroenterology. 2005;129:50–65. doi:10.1053/j.gastro.2005.05.013. 16012934

[33] Kasran A, Boon L, Wortel CH, Hogezand RA, Schreiber S, Goldin E, Boer M, Geboes K, Rutgeerts P, Ceuppens JL. Safety and tolerability of antagonist anti-human CD40 Mab ch5D12 in patients with moderate to severe Crohn’s disease. Aliment Pharmacol Ther. 2005;22:111–122. doi:10.1111/j.1365-2036.2005.02526.x. 16011669

[34] Caton ML, Smith-Raska MR, Reizis B. Notch-RBP-J signaling controls the homeostasis of CD8- dendritic cells in the spleen. J Exp Med. 2007;204:1653–1664. doi:10.1084/jem.20062648. 17591855 PMC2118632

[35] Homig-Holzel C, Hojer C, Rastelli J, Casola S, Strobl LJ, Muller W, Quintanilla-Martinez L, Gewies A, Ruland J, Rajewsky K, et al. Constitutive CD40 signaling in B cells selectively activates the noncanonical NF-kappaB pathway and promotes lymphomagenesis. J Exp Med. 2008;205:1317–1329. doi:10.1084/jem.20080238. 18490492 PMC2413030

[36] Chassaing B, Srinivasan G, Delgado MA, Young AN, Gewirtz AT, Vijay-Kumar M. Fecal lipocalin 2, a sensitive and broadly dynamic non-invasive biomarker for intestinal inflammation. PLoS One. 2012;7:e44328. doi:10.1371/journal.pone.0044328. 22957064 PMC3434182

[37] Brandwein SL, McCabe RP, Cong Y, Waites KB, Ridwan BU, Dean PA, Ohkusa T, Birkenmeier EH, Sundberg JP, Elson CO. Spontaneously colitic C3H/HeJBir mice demonstrate selective antibody reactivity to antigens of the enteric bacterial flora. J Immunol. 1997;159:44–52. https://www.ncbi.nlm.nih.gov/pubmed/9200437. 9200437

[38] Suerbaum S, Josenhans C, Sterzenbach T, Drescher B, Brandt P, Bell M, Droge M, Fartmann B, Fischer HP, Ge Z, et al. The complete genome sequence of the carcinogenic bacterium Helicobacter hepaticus. Proc Natl Acad Sci U S A. 2003;100:7901–7906. doi:10.1073/pnas.1332093100. 12810954 PMC164685

[39] Ward JM, Anver MR, Haines DC, Benveniste RE. Chronic active hepatitis in mice caused by Helicobacter hepaticus. Am J Pathol. 1994;145:959–968. https://www.ncbi.nlm.nih.gov/pubmed/7943185. 7943185 PMC1887338

[40] Whary MT, Morgan TJ, Dangler CA, Gaudes KJ, Taylor NS, Fox JG. Chronic active hepatitis induced by Helicobacter hepaticus in the A/JCr mouse is associated with a Th1 cell-mediated immune response. Infect Immun. 1998;66:3142–3148. doi:10.1128/IAI.66.7.3142-3148.1998 9632578 PMC108325

[41] Boog CJ, de Graeff-meeder ER, Lucassen MA, van der Zee R, Voorhorst-Ogink MM, van Kooten PJ, Geuze HJ, van Eden W. Two monoclonal antibodies generated against human hsp60 show reactivity with synovial membranes of patients with juvenile chronic arthritis. J Exp Med. 1992;175:1805–1810. doi:10.1084/jem.175.6.1805 1316935 PMC2119253

[42] Lupp C, Robertson ML, Wickham ME, Sekirov I, Champion OL, Gaynor EC, Finlay BB. Host-mediated inflammation disrupts the intestinal microbiota and promotes the overgrowth of Enterobacteriaceae. Cell Host Microbe. 2007;2:119–129. doi:10.1016/j.chom.2007.06.010. 18005726

[43] Stecher B, Robbiani R, Walker AW, Westendorf AM, Barthel M, Kremer M, Chaffron S, Macpherson AJ, Buer J, Parkhill J, et al. Salmonella enterica serovar typhimurium exploits inflammation to compete with the intestinal microbiota. PLoS Biol. 2007;5:2177–2189. doi:10.1371/journal.pbio.0050244. 17760501 PMC1951780

[44] Konrad A, Cong Y, Duck W, Borlaza R, Elson CO. Tight mucosal compartmentation of the murine immune response to antigens of the enteric microbiota. Gastroenterology. 2006;130:2050–2059. doi:10.1053/j.gastro.2006.02.055. 16762628

[45] Pabst O, Slack E. IgA and the intestinal microbiota: the importance of being specific. Mucosal Immunol. 2020;13:12–21. doi:10.1038/s41385-019-0227-4. 31740744 PMC6914667

[46] Kawamoto S, Tran TH, Maruya M, Suzuki K, Doi Y, Tsutsui Y, Kato LM, Fagarasan S. The inhibitory receptor PD-1 regulates IgA selection and bacterial composition in the gut. Science. 2012;336:485–489. doi:10.1126/science.1217718. 22539724

[47] Palm NW, de Zoete MR, Cullen TW, Barry NA, Stefanowski J, Hao L, Degnan PH, Hu J, Peter I, Zhang W, et al. Immunoglobulin A coating identifies colitogenic bacteria in inflammatory bowel disease. Cell. 2014;158:1000–1010. doi:10.1016/j.cell.2014.08.006. 25171403 PMC4174347

[48] Zimmermann K, Haas A, Oxenius A. Systemic antibody responses to gut microbes in health and disease. Gut Microbes. 2012;3:42–47. doi:10.4161/gmic.19344. 22356852

[49] Wilmore JR, Gaudette BT, Gomez Atria D, Hashemi T, Jones DD, Gardner CA, Cole SD, Misic AM, Beiting DP, Allman D. Commensal microbes induce serum IgA responses that protect against polymicrobial sepsis. Cell Host Microbe. 2018;23:302–311 e303. doi:10.1016/j.chom.2018.01.005. 29478774 PMC6350773

[50] Furrie E, Macfarlane S, Cummings JH, Macfarlane GT. Systemic antibodies towards mucosal bacteria in ulcerative colitis and Crohn’s disease differentially activate the innate immune response. Gut. 2004;53:91–98. doi:10.1136/gut.53.1.91. 14684582 PMC1773925

[51] Dotan I. New serologic markers for inflammatory bowel disease diagnosis. Dig Dis. 2010;28:418–423. doi:10.1159/000320396. 20926866

[52] Gonzalez-Lopez MA, Velazquez-Guadarrama N, Romero-Espejel ME, Olivares-Trejo Jde J. Helicobacter pylori secretes the chaperonin GroEL (HSP60), which binds iron. FEBS Lett. 2013;587:1823–1828. doi:10.1016/j.febslet.2013.04.048. 23684642

[53] Noll A, Autenrieth IB. Immunity against Yersinia enterocolitica by vaccination with Yersinia HSP60 immunostimulating complexes or Yersinia HSP60 plus interleukin-12. Infect Immun. 1996;64:2955–2961. doi:10.1128/IAI.64.8.2955-2961.1996 8757820 PMC174174

[54] Young D, Lathigra R, Hendrix R, Sweetser D, Young RA. Stress proteins are immune targets in leprosy and tuberculosis. Proc Natl Acad Sci U S A. 1988;85:4267–4270. doi:10.1073/pnas.85.12.4267. 3132709 PMC280408

[55] Bulut Y, Michelsen KS, Hayrapetian L, Naiki Y, Spallek R, Singh M, Arditi M. Mycobacterium tuberculosis heat shock proteins use diverse Toll-like receptor pathways to activate pro-inflammatory signals. J Biol Chem. 2005;280:20961–20967. doi:10.1074/jbc.M411379200. 15809303

[56] Tanaka A, Kamada T, Yokota K, Shiotani A, Hata J, Oguma K, Haruma K. Helicobacter pylori heat shock protein 60 antibodies are associated with gastric cancer. Pathol Res Pract. 2009;205:690–694. doi:10.1016/j.prp.2009.04.008. 19450936

[57] Xu M, Pokrovskii M, Ding Y, Yi R, Au C, Harrison OJ, Galan C, Belkaid Y, Bonneau R, Littman DR. c-MAF-dependent regulatory T cells mediate immunological tolerance to a gut pathobiont. Nature. 2018;554:373–377. doi:10.1038/nature25500. 29414937 PMC5814346

[58] Kullberg MC, Jankovic D, Feng CG, Hue S, Gorelick PL, McKenzie BS, Cua DJ, Powrie F, Cheever AW, Maloy KJ, et al. IL-23 plays a key role in Helicobacter hepaticus-induced T cell-dependent colitis. J Exp Med. 2006;203:2485–2494. doi:10.1084/jem.20061082. 17030948 PMC2118119

[59] Fox JG, Dewhirst FE, Tully JG, Paster BJ, Yan L, Taylor NS, Collins MJ Jr., Gorelick PL, Ward JM. Helicobacter hepaticus sp. nov., a microaerophilic bacterium isolated from livers and intestinal mucosal scrapings from mice. J Clin Microbiol. 1994;32:1238–1245. doi:10.1128/JCM.32.5.1238-1245.1994 8051250 PMC263656

[60] Nagalingam NA, Robinson CJ, Bergin IL, Eaton KA, Huffnagle GB, Young VB. The effects of intestinal microbial community structure on disease manifestation in IL-10-/- mice infected with Helicobacter hepaticus. Microbiome. 2013;1:15. doi:10.1186/2049-2618-1-15. 24450737 PMC3971628

[61] Beckwith CS, Franklin CL, Hook RR Jr., Besch-Williford CL, Riley LK. Fecal PCR assay for diagnosis of Helicobacter infection in laboratory rodents. J Clin Microbiol. 1997;35:1620–1623. doi:10.1128/JCM.35.6.1620-1623.1997 9163500 PMC229805

[62] Franklin CL, Gorelick PL, Riley LK, Dewhirst FE, Livingston RS, Ward JM, Beckwith CS, Fox JG. Helicobacter typhlonius sp. nov., a novel murine urease-negative helicobacter species. J Clin Microbiol. 2001;39:3920–3926. doi:10.1128/JCM.39.11.3920-3926.2001. 11682508 PMC88465

[63] Shen Z, Fox JG, Dewhirst FE, Paster BJ, Foltz CJ, Yan L, Shames B, Perry L. Helicobacter rodentium sp. nov., a urease-negative Helicobacter species isolated from laboratory mice. Int J Syst Bacteriol. 1997;47:627–634. doi:10.1099/00207713-47-3-627. 9226892

[64] Fox JG, Yan LL, Dewhirst FE, Paster BJ, Shames B, Murphy JC, Hayward A, Belcher JC, Mendes EN. Helicobacter bilis sp. nov., a novel Helicobacter species isolated from bile, livers, and intestines of aged, inbred mice. J Clin Microbiol. 1995;33:445–454. doi:10.1128/JCM.33.2.445-454.1995 7536217 PMC227964

[65] Schloss PD, Westcott SL, Ryabin T, Hall JR, Hartmann M, Hollister EB, Lesniewski RA, Oakley BB, Parks DH, Robinson CJ, et al. Introducing mothur: open-source, platform-independent, community-supported software for describing and comparing microbial communities. Appl Environ Microbiol. 2009;75:7537–7541. doi:10.1128/AEM.01541-09. 19801464 PMC2786419

[66] Mandal S, Van Treuren W, White RA, Eggesbo M, Knight R, Peddada SD. Analysis of composition of microbiomes: a novel method for studying microbial composition. Microb Ecol Health Dis. 2015;26:27663. doi:10.3402/mehd.v26.27663. 26028277 PMC4450248

