# Supplementary material for: Helicobacter hepaticus is required for immune targeting of bacterial heat shock protein 60 and fatal colitis in mice
Source: Gut Microbes. 2021 Feb 8;13(1):1882928. doi: 10.1080/19490976.2021.1882928 (PMC7889221; doi:10.1080/19490976.2021.1882928)
Supplement: Supplemental Material [file KGMI_A_1882928_SM6325.zip › Supplementary information/201210_Friedrich_et_al_Suppl_legend_rev.docx]

**Supplemental Material**

**Figure S1. Intensities of Ig-related proteins (related to Figure 2(a,b)).** Experiment was performed as in Figure 2(a,b), but data was filtered for Ig-related proteins only. Intensities of all Ig-related proteins detected by LC-MS/MS within every single control or DC-LMP1/CD40 sample are illustrated by bar graphs as mean ± SEM (two pooled experiments, n=6).

**Figure S2. Screening of mice for colonization with *Helicobacter* (related to Figure 3(a-c).** Bacterial DNA was extracted from fecal samples from Ctr or DC-LMP1/CD40 mice before (*Hh*-pos, upper panel) and after rendering them *Hh*-free (lower panel) at the indicated time points. 16S rRNA gene primers were used to detect the species indicated and amplicons were analyzed by agarose gel electrophoresis (n=5-14). bac: universal bacteria; *Hspp: Helicobacter* species; *Hh*: *H. hepaticus*; *Ht*: *H. typhlonius*; *Hr*: *H. rodentium*; *Hb*: *H.bilis*

**Figure S3. Screening of mice for colonization with *Helicobacter* upon infection (related to Figure 5(b).** Bacterial DNA was extracted from fecal samples from PBS-treated Ctr or DC-LMP1/CD40 mice and from animals inoculated with *Hh* at the indicated time points. 16S rRNA gene primers were used to detect the species indicated and amplicons were analyzed by agarose gel electrophoresis. Shown is one representative experiment out of two (n=4). bac: universal bacteria; *Hspp*: *Helicobacter* species; *Hh*: *H. hepaticu*s; *Ht*: *H. typhlonius*; *Hr*: *H. rodentium*; *Hb*: *H. bilis*

**Figure S4. Loss of intestinal CD103^+^ DCs and iTregs in 25-week-old *Hh*-free DC-LMP1/CD40 mice (related to Figure 6(a,b)).** (a-b) Different cell subsets in the colonic LP were analyzed in 25-week-old *Hh*-free Ctr and DC-LMP1/CD40 animals. Shown are representative FACS-plots as well as pooled statistics from two experiments (mean ± SEM, n=7-8), illustrating frequencies of the indicated cell subsets. (a) DCs were gated on single, live, CD45^+^, lymphocytes, MHCII^+^CD11c^+^, CD64^-^ cells. (b) Tregs were gated on single, live, CD45^+^, CD3^+^CD4^+^, FoxP3^+^CD25^+^, Helios^-^ (iTregs) or Helios^+^ (nTregs).

**Figure S5. Flow cytometry gating strategies for DCs, Tregs and effector T cells (related to Figure 6 and S4).** Gating strategies for (a) DCs in the colonic Lamina Propria (b) Tregs in the colonic Lamina Propria and (c) CD4^+^ T cell subsets in the colonic Lamina Propria.
