# Supplementary material for: Helicobacter hepaticus is required for immune targeting of bacterial heat shock protein 60 and fatal colitis in mice
Source: Gut Microbes. 2021 Feb 8;13(1):1882928. doi: 10.1080/19490976.2021.1882928 (PMC7889221; doi:10.1080/19490976.2021.1882928)

# Figure S5

(a) gating strategy for DCs in the colonic Lamina Propria (single cells, live, CD45$^+$, CD11c$^+$MHC-II$^+$, CD64$^-$)

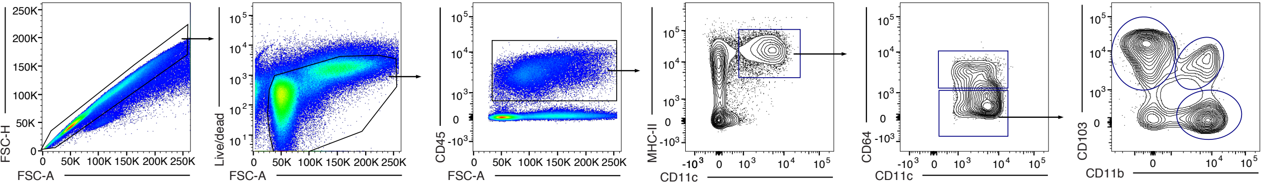


(b) gating strategy for Tregs in the colonic Lamina Propria (single cells, live, CD45$^+$, CD3$^+$CD4$^+$, CD25$^+$Foxp3$^+$)

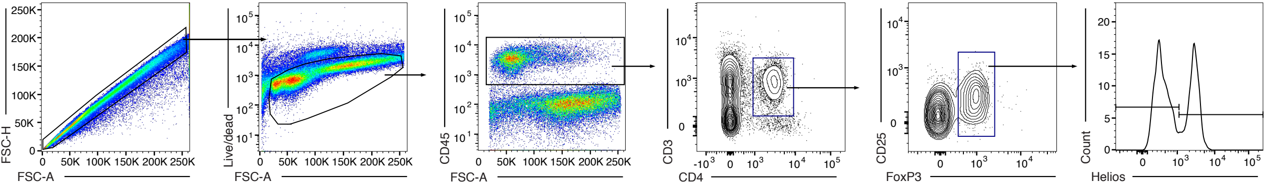


(b) gating strategy for effector T cells in the colonic Lamina Propria (single cells, live, CD45$^+$, CD3$^+$CD4$^+$)

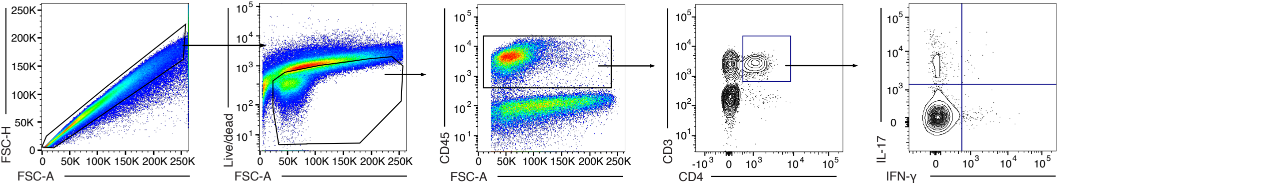

Supplement: Supplemental Material [file KGMI_A_1882928_SM6325.zip › Supplementary information/Figure_S5.pdf]
