# Supplementary figures and images for: Helicobacter hepaticus is required for immune targeting of bacterial heat shock protein 60 and fatal colitis in mice
Source: Gut Microbes. 2021 Feb 8;13(1):1882928. doi: 10.1080/19490976.2021.1882928 (PMC7889221; doi:10.1080/19490976.2021.1882928)

# Figure S1

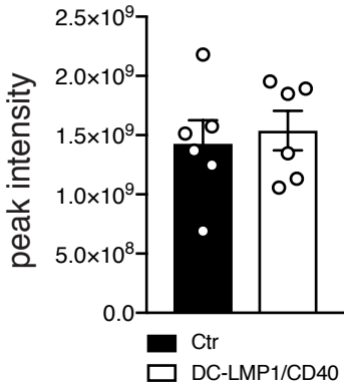

Supplement: Supplemental Material [file KGMI_A_1882928_SM6325.zip › Supplementary information/Figure_S1.pdf]

**Figure S2**

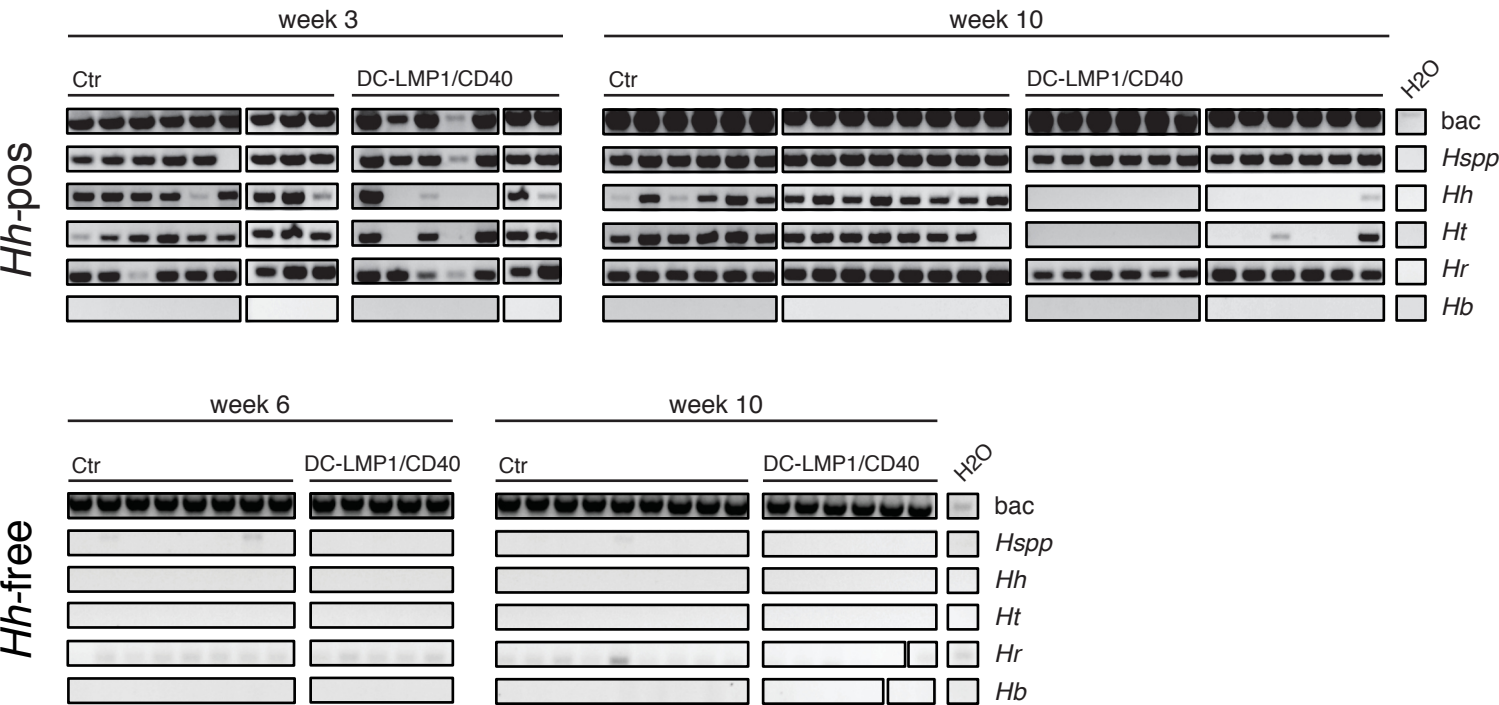

Supplement: Supplemental Material [file KGMI_A_1882928_SM6325.zip › Supplementary information/Figure_S2.pdf]

**Figure S3**

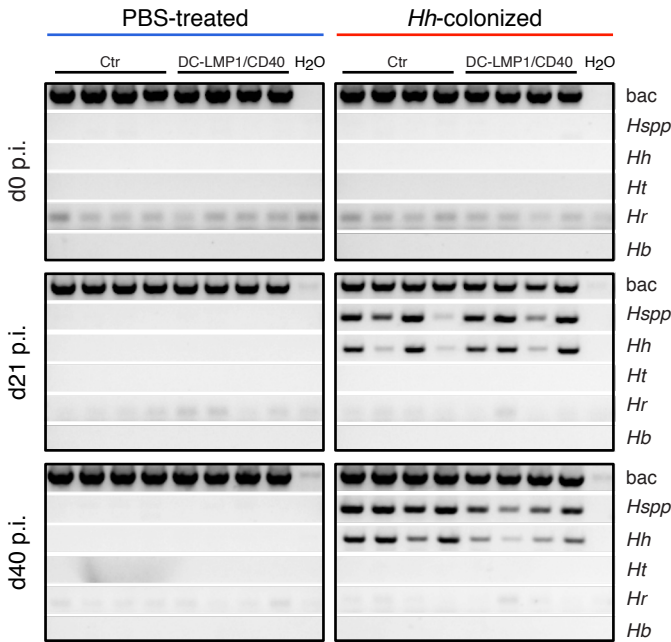

Supplement: Supplemental Material [file KGMI_A_1882928_SM6325.zip › Supplementary information/Figure_S3.pdf]

**Figure S4**

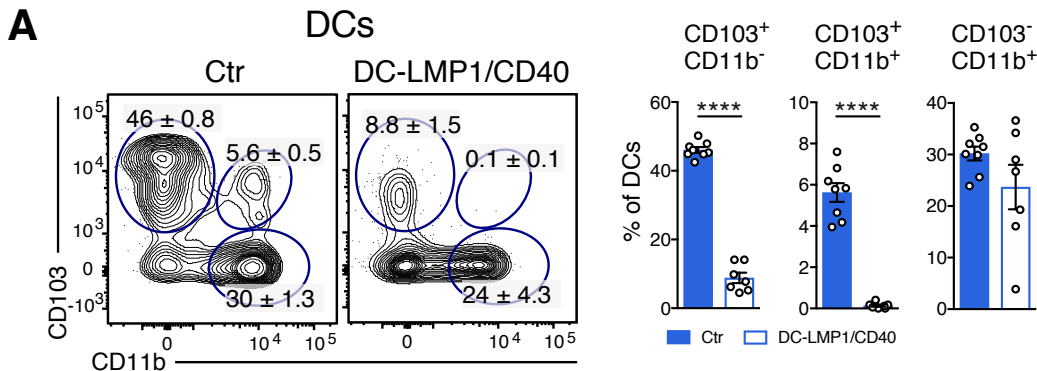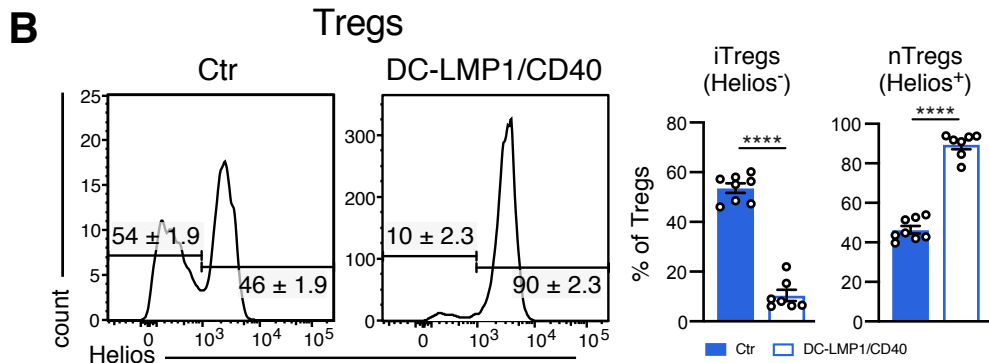

Supplement: Supplemental Material [file KGMI_A_1882928_SM6325.zip › Supplementary information/Figure_S4.pdf]
